# Comparative effectiveness of alternative second-line oral glucose-lowering therapies for type 2 diabetes: a precision medicine approach applied to routine data

**DOI:** 10.1007/s00125-025-06447-x

**Published:** 2025-05-31

**Authors:** Stephen O’Neill, Patrick Bidulka, David G. Lugo-Palacios, Orlagh Carroll, Ignacio Leiva-Escobar, Richard Silverwood, Andrew Briggs, Amanda I. Adler, Kamlesh Khunti, Richard Grieve

**Affiliations:** 1 Department of Health Services Research and Policy, London School of Hygiene & Tropical Medicine, London, UK; 2 Department of Non-Communicable Disease Epidemiology, London School of Hygiene & Tropical Medicine, London, UK; 3 Department of Infectious Disease Epidemiology & International Health, London School of Hygiene & Tropical Medicine, London, UK; 4 Heidelberg University, Medical Faculty of Heidelberg, Internal Medicine IX - Department of Clinical Pharmacology and Pharmacoepidemiology, Heidelberg University Hospital, Heidelberg, Germany; 5 Centre for Longitudinal Studies, UCL Social Research Institute, University College London, London, UK; 6 Diabetes Trials Unit, The Oxford Centre for Diabetes, Endocrinology and Metabolism, University of Oxford, OCDEM Building Churchill Hospital, Headington, UK; 7 Diabetes Research Centre, University of Leicester, Leicester, UK

**Keywords:** Diabetes mellitus, Dipeptidyl peptidase-4 inhibitors (DPP4i), Electronic health records, Heterogeneous treatment effects, Instrumental variables, Multiple long-term conditions (MLTCs), Precision medicine, Sodium–glucose cotransporter-2 inhibitors (SGLT2i), Sulfonylureas (SU)

## Abstract

**Aims/hypothesis:**

National clinical guidelines recommend that second-line treatment for type 2 diabetes mellitus is chosen according to individuals’ characteristics but there is limited evidence available to inform this choice. This paper’s aim is to compare the effects on HbA_1c_ of sulfonylureas (SU), dipeptidyl peptidase-4 inhibitors (DPP4i) or sodium–glucose cotransporter-2 inhibitors (SGLT2i) added to metformin as second-line oral glucose-lowering treatments according to an individual’s age, baseline HbA_1c_ and presence of multiple long-term conditions (MLTCs).

**Methods:**

We accessed primary care-hospital linked data for 41,790 individuals from the Clinical Practice Research Datalink (CPRD) in England who initiated second-line treatment after metformin between 2015 and 2021. We combined target trial emulation with instrumental variable analysis to reduce the risk of confounding. The outcome was change in HbA_1c_ between baseline and 1 year follow-up. We reported results stratified by age (18–49 years, 50–69 years and ≥70 years), baseline HbA_1c_ (<67 mmol/mol [<8.3%], 67–77 mmol/mol [8.3–9.2%] and >77 mmol/mol [>9.2%]) and presence of MLTCs.

**Results:**

The mean (95% CI) difference in HbA_1c_ change for SGLT2i vs SU was larger for people aged 18–49 years (−5.74 mmol/mol [−7.47, −4.01]) (−0.5% [−0.7, −0.4]) than for those aged 50–69 years (−4.03 mmol/mol [−5.61, −2.44]) (−0.4% [−0.5, −0.2]) and for those aged 70 years or over (−2.68 mmol/mol [−4.50, −0.86]) (−0.3% [−0.4, −0.07]). The mean (95% CI) difference in HbA_1c_ change for SGLT2i vs DPP4i was −5.80 mmol/mol (−7.60, −4.00) (−0.5% [−0.7, −0.4]) for those aged 18–49 years, −4.13 mmol/mol (−5.82, −2.45) (−0.4% [−0.5, −0.2]) for those aged 50–69 years and −3.13 mmol/mol (−5.01, −1.24) (−0.3% [−0.4, −0.1]) for those aged ≥70 years. The mean difference (improvement) in HbA_1c_ was similar across subgroups defined by baseline HbA_1c_ or presence of MLTCs. For SGLT2i vs SU, the mean (95% CI) difference was −5.37 mmol/mol (−7.13, −3.62) (−0.5% [−0.6, −0.3]) for people without MLTC and −3.72 mmol/mol (−5.34, −2.10]) (−0.3% [−0.5, −0.2]) for people with MLTC. For SGLT2i vs DPP4i the corresponding estimated differences (95% CI) were −5.44 mmol/mol (−7.27, −3.61) (−0.5% [−0.7, −0.3]) for those without MLTC and −3.93 mmol/mol (−5.64, −2.21) (−0.3% [−0.5, −0.2]) for those with MLTC.

**Conclusions/interpretation:**

Second-line treatment with SGLT2i is more effective than SU or DPP4i in reducing HbA_1c_ across subgroups of people defined by age, baseline HbA_1c_ and presence of MLTCs. Our evidence complements RCTs in using routinely available information on demographic characteristics, biomarkers and comorbidities to inform an individualised approach.

**Graphical Abstract:**

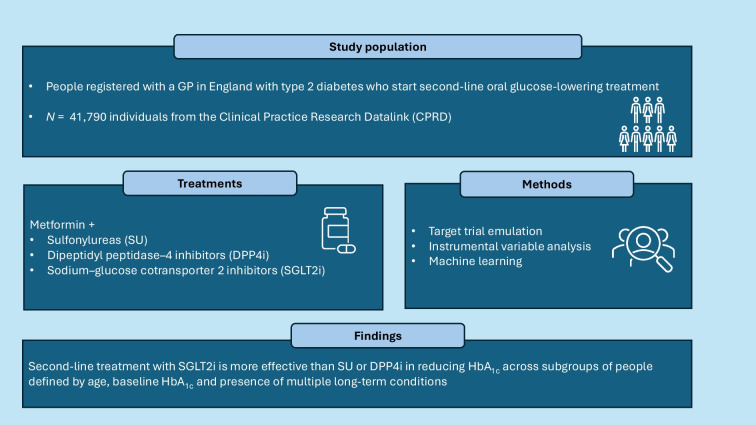

**Supplementary Information:**

The online version contains peer-reviewed but unedited supplementary material available at 10.1007/s00125-025-06447-x.


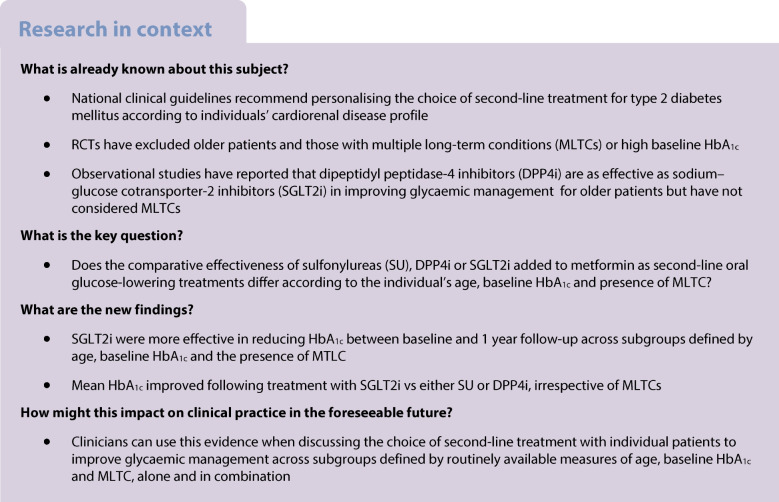


## Introduction

Type 2 diabetes mellitus is a heterogeneous condition with varying underlying pathophysiologies. After first-line oral treatment with metformin monotherapy, the most common second-line treatments globally are sulfonylureas (SU), dipeptidyl peptidase-4 inhibitors (DPP4i) and sodium–glucose cotransporter-2 inhibitors (SGLT2i) [1–3]. National clinical guidelines recommend SGLT2i for people with heart failure, established CVD, at high risk of CVD, or with chronic kidney disease (CKD) [4]. For people who have type 2 diabetes but no specific cardiorenal indications (~70%) [5], National Institute for Health and Care Excellence (NICE) guidelines recommend that the choice of second-line treatment is ‘personalised according to the individual’ and that target HbA_1c_ levels are relaxed for those who are older, frailer or with a reduced life expectancy [4]. The ADA/EASD also recommends a patient-centred approach that recognises comorbidities [6].

RCTs have shown that SGLT2i can improve cardiovascular and kidney outcomes for people at high risk of or with pre-existing CVD and CKD [7–11] but have excluded people with suboptimal glycaemic management in type 2 diabetes (high baseline HbA_1c_) or older people (e.g. over 75 years old) who often have multiple long-term conditions (MLTCs) [12, 13]. Precision medicine requires evidence on the comparative effectiveness of these three treatments in managing blood glucose, according to clinical characteristics that are measured in routine practice [14–16], including age, baseline HbA_1c_ and the presence of MLTC.

Observational studies have investigated clinical characteristics that may modify the relative effectiveness of SGLT2i vs DPP4i in general populations of people with type 2 diabetes. One study found no evidence that the effectiveness of SGLT2i vs DPP4i in improving cardiovascular outcomes differed across baseline levels of HbA_1c_ [17]. Another study reported that increasing age and lower eGFR were associated with better glycaemic response with DPP4i vs SGLT2i [14]. A recent study found similar HbA_1c_ reductions between SGLT2i and DPP4i for people aged over 70 years [18]. However, none of these studies considered SU, a common second-line treatment, or MLTC, which may modify the relative effectiveness of alternative second-line treatments.

The PERMIT study addresses this gap in knowledge by emulating the design of a hypothetical pragmatic RCT in assessing the comparative effectiveness of the three most common second-line oral glucose-lowering drug classes (SU, DPP4i and SGLT2i) for the broad eligible population of people with type 2 diabetes in England [19–21]. The PERMIT study combined routinely collected health data from Clinical Practice Research Datalink (CPRD) [22] with an instrumental variable analysis [23] to reduce the risk of confounding, and reported the causal effects of SGLT2i vs SU or DPP4i on clinical outcomes. The original study reported that, overall, SGLT2i were more effective in reducing HbA_1c_, BMI and systolic BP (SBP) than either SU or DPP4i [20].

This paper’s aims are to assess the comparative effectiveness of SU, DPP4i and SGLT2i added to metformin as second-line oral glucose-lowering treatments according to an individual’s age, baseline HbA_1c_ and presence of MLTC, and to generate hypotheses about whether specific MLTCs combined with age and baseline HbA_1c_ modify comparative effectiveness.

## Methods

### Study design and participants

We applied target trial emulation principles [24] to primary care data from the CPRD [22] to identify people with type 2 diabetes who initiated second-line oral glucose-lowering treatment [20]. The CPRD Aurum database covers a representative sample of approximately 13% of the UK population and includes longitudinal information on primary care diagnoses, prescriptions, demographic information and laboratory test results [22]. We accessed information from linked Hospital Episode Statistics (HES) Admitted Patient Care database on diagnoses, procedures, sociodemographic characteristics and admission and discharge dates [25]. We included individuals with type 2 diabetes aged 18 years and over registered with a GP in England who intensified treatment from first- to second-line oral glucose-lowering treatments between 1 January 2015 and 31 December 2020 with a first-ever prescription of SU, DPP4i or SGLT2i, added to metformin [20]. See also electronic supplementary materials (ESM) Methods.

### Treatments

We included people prescribed one of SU, DPP4i or SGLT2i, added to metformin monotherapy, which accounted for over 90% of the second-line treatments prescribed and is recommended by NICE guidelines [1, 2, 4].

### Outcomes

The primary outcome was the mean absolute change in HbA_1c_ between baseline and 1 year following second-line treatment prescription (HbA_1c_ at 1 year − HbA_1c_ at baseline). We used the measurement closest to the 1 year follow-up timepoint and allowed for measures within ±90 days, otherwise the measure was designated as missing.

### Covariates

We defined baseline HbA_1c_, SBP and diastolic BP (DBP), eGFR and BMI [26], patient sociodemographic characteristics including biological sex, total number of people registered at the individual’s GP, smoking and alcohol status, relevant co-prescriptions, past hospitalisations and long-term conditions (LTCs) recorded at baseline (see ESM Methods). The choice of LTCs were informed by recommendations from a Delphi study [27] and were collated in the CPRD–HES linked data. The LTCs were classified into concordant (classical complications of diabetes) and discordant MLTCs (all other LTCs) [28] as follows:

- Concordant LTC: atrial fibrillation, CKD, clinical hypertension, blindness, CVD (myocardial infarction, ischaemic heart disease, heart failure, stroke, unstable angina, and peripheral artery disease) and previous lower extremity amputation
- Discordant LTC: asthma, cancer (any), chronic obstructive pulmonary disease (COPD), clinical depression, dementia, epilepsy, irritable bowel syndrome, neurological disorders, HIV infection, liver disease, rheumatoid arthritis, severe mental illness, thyroid disease and valvular heart disease

### Identifying MLTCs and demographic profiles

We defined profiles as all combinations of the 21 LTCs and three characteristics: sex (male or female); age (18–59 years, 60–69 years and ≥70 years); and HbA_1c_ (<67 mmol/mol [<8.3%], 67–77 mmol/mol [8.3–9.2%] and >77 mmol/mol [>9.2%]). As it was not feasible to assess comparative effectiveness for all profiles, we selected profiles as follows. First, we assigned individuals to groups defined by profile of LTCs and demographic characteristics, and ordered profiles according to the number of individuals (i.e. descending prevalence). Second, we retained profiles (*n*=64) for which the number of individuals exceeded a threshold of *n*=100. We chose this threshold to balance the need for precise estimates of comparative effectiveness according to profile, with the requirement for enough profiles (29,067 out of 41,790, or 70%). The remaining individuals were included as the base group. We estimated subgroup effects for each profile by aggregating individual-level effect estimates, which required each individual to belong to one profile (see below).

### Statistical analysis

We reported estimated differences between treatments for the change in HbA_1c_ from baseline to 1 year follow-up, along with 95% CIs. We contrasted these estimated differences for pairs of subgroups (e.g. people aged 50–69 years vs 18–49 years) to obtain what we termed ‘difference in differences’ estimates that estimate the magnitude of differences between subgroups in the between-treatment changes in HbA_1c_ from baseline to 1 year follow-up. We report tests of the null hypothesis that the between-treatment change was the same for pairs of subgroups (i.e. that the ‘difference in differences’ for HbA_1c_ change was 0). We did not adjust for multiple comparisons. All analyses were conducted in Stata 16.1 SE (StataCorp), with a two-tailed *p* value <0.05 denoting statistical significance.

We followed previous pharmaco-epidemiological research and the main PERMIT study [20] in using clinician preference as an instrument for treatment prescribed to reduce the risk of bias due to confounding by indication (see ESM Methods for details) [29, 30]. The instrumental variable approach taken was the two-stage residual inclusion (2SRI) method [31] with recycled predictions [32], which enabled us to estimate comparative effectiveness for each individual while reducing the risk of bias from unmeasured confounding under the assumptions that the treatment assignment model and outcome regression were correctly specified and the instrumental variable was valid. To aid model specification we applied post-double-selection [33] of variables for inclusion with adaptive least absolute shrinkage and selection operator (LASSO), a machine learning approach [34].

Primary care providers in England (GPs) were grouped into clinical commissioning groups (CCGs) during the study period, and these informed prescribing decisions, with considerable variation at CCG level in the second-line glucose-lowering therapy prescribed (ESM Fig. 1). The first-stage model estimated the probability that each person was prescribed each treatment given their baseline covariates and their CCG’s tendency to prescribe (TTP) that treatment [35]. The second-stage outcome model included generalised residuals from the first-stage (propensity score) model. The outcome (change in HbA_1c_ at 1 year) model was estimated by ordinary least squares (OLS), while the treatment assignment model was a multinomial logit. Variables for each model and the choice of effect modifiers were selected by LASSO regression [36, 37] (see ESM Methods).

### Missing data

Data were missing for the outcome and baseline covariates (ethnicity, Index of Multiple Deprivation, HbA_1c_, SBP, DBP, BMI, eGFR, smoking and alcohol status) if these measures were not recorded by the GP within the requisite time period. We undertook complete case analysis, which our previous research found gave similar results to multiple imputation in the same study population [20].

## Results

### Cohort description

A total of 41,790 individuals met the inclusion criteria (ESM Fig. 2). Table 1 reports baseline characteristics stratified by the second-line oral glucose-lowering therapy prescribed. The mean age of those prescribed SGLT2i (56.1 years, SD 10.4) was lower than those prescribed DPP4i (62.0 years, SD 12.2 ) or SU (60.3 years, SD 12.2) (Table 1). The mean baseline HbA_1c_ was higher for the group prescribed SU (80.4 mmol/mol, SD 21.7 [9.5%, SD 4.1]) than for those prescribed DPP4i (71.0 mmol/mol, SD 15.3 [8.6%, SD 3.5]) or SGLT2i (73.9 mmol/mol, SD 16.9 [8.9%, SD 3.7]). Only 18.7% did not have any other LTC (ESM Table 1).

**Table 1:** Baseline characteristics of the primary/secondary care-linked study population, stratified by the second-line glucose-lowering therapies prescribed

Characteristic	SU	DPP4i	SGLT2i
*N* (% of total study population)	14,059 (33.6)	19,907 (47.6)	7824 (18.7)
Female sex	5541 (39.4)	7818 (39.3)	3176 (40.6)
Age in years, mean (SD)	60.3 (12.2)	62.0 (12.2)	56.1 (10.4)
Ethnicity			
White	10,945 (77.9)	16,107 (80.9)	6453 (82.5)
South Asian	1962 (14)	2653 (13.3)	993 (12.7)
Black, Mixed or Other	1152 (8.2)	1147 (5.8)	378 (4.8)
Index of Multiple Deprivation quintile^a^			
1 (least deprived)	1988 (14.1)	2971 (14.9)	1330 (17.0)
2	2445 (17.4)	3620 (18.2)	1441 (18.4)
3	2712 (19.3)	3753 (18.9)	1464 (18.7)
4	3349 (23.8)	4443 (22.3)	1704 (21.8)
5 (most deprived)	3559 (25.3)	5108 (25.7)	1883 (24.1)
Year of initiating second-line glucose-lowering therapy			
2015	4086 (29.1)	3070 (15.4)	709 (9.1)
2016	3063 (21.8)	3846 (19.3)	916 (11.7)
2017	2470 (17.6)	4083 (20.5)	1316 (16.8)
2018	2100 (14.9)	4323 (21.7)	1715 (21.9)
2019	1418 (10.1)	2908 (14.6)	1820 (23.3)
2020	922 (6.6)	1677 (8.4)	1348 (17.2)
No. of years on first-line treatment (metformin monotherapy), median (IQR)	4.8 (2.1–8.3)	5.6 (2.9–9.3)	4.3 (2.1–7.6)
General Practice size, mean number of patients registered, median (IQR)	9632 (6158–13,355)	9923 (6508–13,688)	10,069 (6843–13,513)
Last HbA_1c_ value (mmol/mol) recorded prior to index date, mean (SD)	80.4 (21.7)	71 (15.3)	73.9 (16.9)
Last HbA_1c_ value (%) recorded prior to index date, mean (SD)	9.5 (4.1)	8.6 (3.5)	8.9 (3.7)
Last HbA_1c_ value recorded prior to index date			
< 65 mmol/mol (< 8.1%)	3691 (26.3)	8180 (41.1)	2690 (34.4)
65–77 mmol/mol (8.1–9.2%)	4122 (29.3)	6869 (34.5)	2563 (32.8)
> 77 mmol/mol (> 9.2%)	6246 (44.4)	4858 (24.4)	2571 (32.9)
Last SBP measure (mmHg) recorded prior to index date, mean (SD)	131.6 (13.9)	131.9 (13.5)	132.2 (13.4)
Last DBP measure (mmHg) recorded prior to index date, mean (SD)	77.7 (9.0)	77.2 (8.9)	79.4 (8.7)
BP status, based on last recorded measure			
Normotensive	3959 (28.2)	5488 (27.6)	1893 (24.2)
Hypertensive^a^	10,090 (71.8)	14,415 (72.4)	5929 (75.8)
Last recorded BMI (kg/m^2^) prior to second-line treatment initiation, mean (SD)	31.5 (6.4)	32.3 (6.5)	35.1 (6.9)
BMI category			
Under/normal weight (<25 kg/m^2^)	1449 (10.3)	1568 (7.9)	200 (2.6)
Overweight (25–30 kg/m^2^)	4457 (31.7)	5887 (29.6)	1404 (17.9)
Obese (> 30 kg/m^2^)	8153 (58)	12,452 (62.6)	6220 (79.5)
Last recorded eGFR (ml/min per 1.73 m^2^) prior to second-line treatment initiation, mean (SD)	90.9 (18.3)	88.5 (18.9)	96.9 (14.2)
eGFR category^a^			
Stage 1–2 (eGFR≥60 ml/min per 1.73 m^2^)	12,782 (93.2)	17,866 (90.8)	7658 (99.2)
Stage 3a–3b (eGFR 30–59 ml/min per 1.73 m^2^)	927 (6.8)	1804 (9.2)	64 (0.8)
MLTCs			
CVD composite	3053 (21.7)	4630 (23.3)	1343 (17.2)
Heart failure	731 (5.2)	1102 (5.5)	269 (3.4)
Previous myocardial infarction	820 (5.8)	1251 (6.3)	408 (5.2)
Previous stroke	717 (5.1)	933 (4.7)	269 (3.4)
Ischaemic heart disease	2378 (16.9)	3743 (18.8)	1084 (13.9)
Unstable angina	389 (2.8)	648 (3.3)	178 (2.3)
Previous lower extremity amputation	108 (0.8)	145 (0.7)	34 (0.4)
History of cancer	2327 (16.6)	3231 (16.2)	798 (10.2)
Blindness	241 (1.7)	294 (1.5)	71 (0.9)
Previous hypoglycaemia	115 (0.8)	160 (0.8)	57 (0.7)
Dementia	230 (1.6)	319 (1.6)	47 (0.6)
Hypertension	8604 (61.2)	12,932 (65)	4656 (59.5)
Clinical depression	2614 (18.6)	3402 (17.1)	1696 (21.7)
Thyroid disease	1306 (9.3)	1849 (9.3)	689 (8.8)
COPD	1330 (9.5)	1767 (8.9)	535 (6.8)
Atrial fibrillation	868 (6.2)	1485 (7.5)	327 (4.2)
Asthma	601 (4.3)	751 (3.8)	369 (4.7)
Liver disease	584 (4.2)	692 (3.5)	319 (4.1)
Neurological disorders	538 (3.8)	718 (3.6)	130 (1.7)
Valvular heart disease	401 (2.9)	647 (3.3)	141 (1.8)
Peripheral arterial disease	320 (2.3)	494 (2.5)	110 (1.4)
Epilepsy	254 (1.8)	316 (1.6)	121 (1.5)
Rheumatoid arthritis	238 (1.7)	318 (1.6)	108 (1.4)
Irritable bowel syndrome	197 (1.4)	260 (1.3)	82 (1.0)
Severe mental illness	374 (2.7)	461 (2.3)	190 (2.4)
HIV infection	38 (0.3)	33 (0.2)	9 (0.1)
Co-prescriptions			
Renin–angiotensin system inhibitor	9978 (71)	14,985 (75.3)	5533 (70.7)
Statin	7029 (50)	11,028 (55.4)	4094 (52.3)
Smoking status			
Non-smoker	3064 (21.8)	4185 (21)	1742 (22.3)
Ex-smoker	7050 (50.1)	10,673 (53.6)	4047 (51.7)
Current smoker	3945 (28.1)	5049 (25.4)	2035 (26)
Alcohol intake			
Non-drinker	1584 (11.3)	1966 (9.9)	781 (10)
Ex-drinker	4169 (29.7)	5917 (29.7)	2150 (27.5)
Current drinker	8306 (59.1)	12,024 (60.4)	4893 (62.5)
NHS region			
North East	970 (6.9)	376 (1.9)	156 (2.0)
North West	2422 (17.2)	4277 (21.5)	1585 (20.3)
Yorkshire and The Humber	499 (3.5)	768 (3.9)	333 (4.3)
East Midlands	465 (3.3)	578 (2.9)	116 (1.5)
West Midlands	1965 (14.0)	3833 (19.3)	1306 (16.7)
East of England	677 (4.8)	798 (4.0)	273 (3.5)
London	3335 (23.7)	3642 (18.3)	1186 (15.2)
South East	2198 (15.6)	3209 (16.1)	1930 (24.7)
South West	1528 (10.9)	2426 (12.2)	939 (12.0)
Hospitalisations in the past year	3671 (26.1)	4665 (23.4)	1592 (20.3)

Data are presented as *n* (column %) unless specified otherwise

^a^*n*=20, 16 and 689 participants were missing IMD quintile, hypertensive status and eGFR category, respectively

*NHS*, National Health Service

**Fig. 1:**
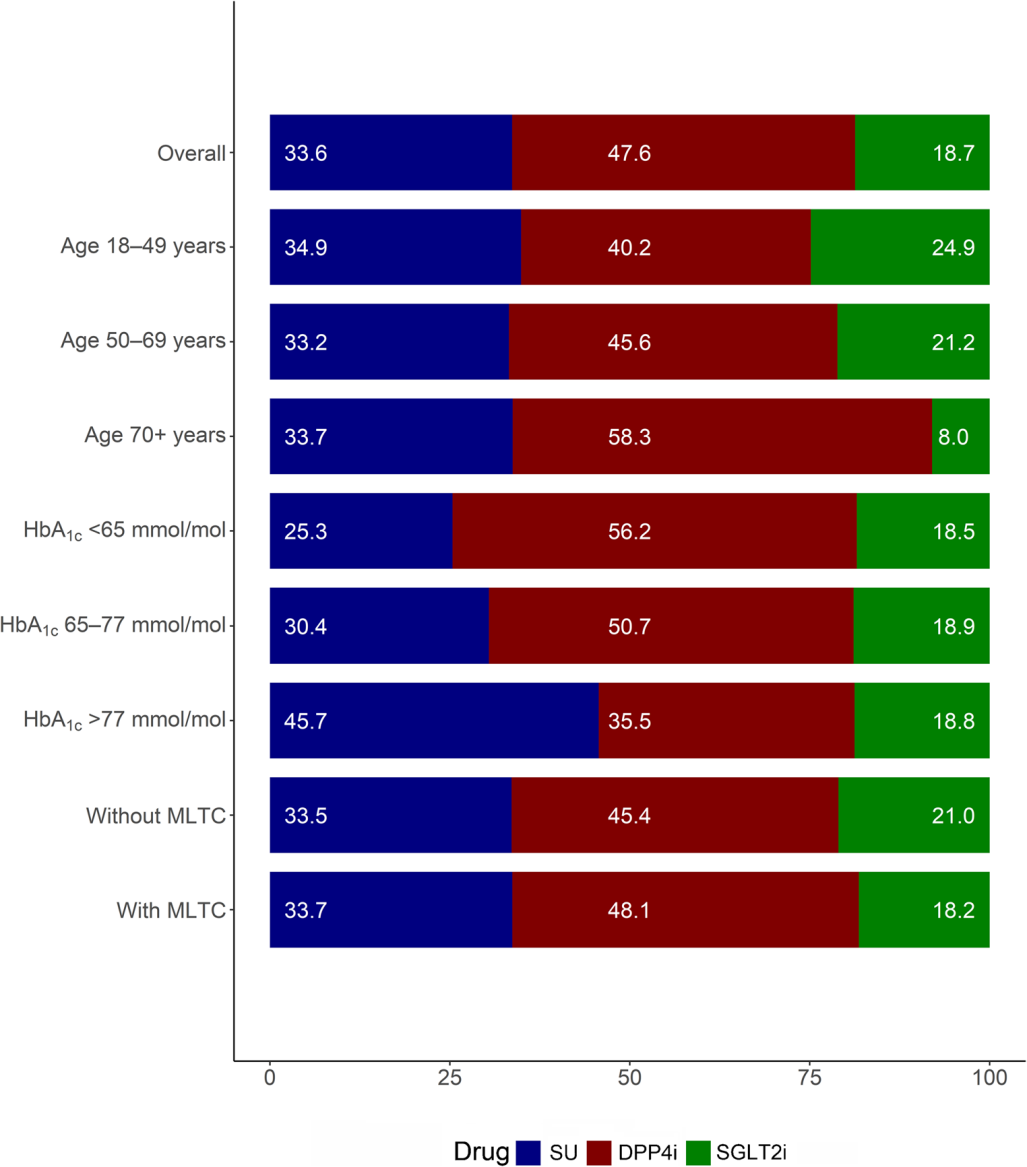
Variation in prescribing of SU, DPP4i and SGLT2i by age, HbA_1c_ and MLTC subgroups

The proportion of individuals prescribed SGLT2i was lower than for those prescribed DPP4i or SU (see Fig. 1). The proportion of individuals prescribed SGLT2i, DPP4i and SU, respectively, was as follows: 24.9%, 40.2% and 34.9% for those aged 18–49 years; 21.2%, 45.6% and 33.2% for those aged 50–69 years; and 8.0%, 58.3% and 33.7% for those aged ≥70 years. The corresponding proportions among people without MLTC were 21.0% (SGLT2i), 45.4% (DPP4i) and 33.5% (SU), and among people with MLTC were 18.2% (SGLT2i), 48.1% (DPP4i), and 33.7% (SU). While the proportions prescribed SGLT2i increased in years 2019–2020 vs 2015–2018, they were still lower than the alternatives across all the subgroups (ESM Fig. 3–9).

### Assessment of instrumental variable assumptions

The instrumental variable, TTP, met a major requirement for validity in that it was strongly associated with the second-line treatment prescribed. The accompanying Cragg–Donald *F* statistics were 1515 for DPP4i and 1413 for SGLT2i. This met the requirement for a strong instrument, where the *F* statistic should exceed 100 [38]. The instrumental variable met another critical requirement in that the measured potential confounders were balanced across levels of the instrumental variable (assumption 2), aside from time period, which was included within the covariate adjustment of the instrumental variable analysis (see ESM Fig. 10 and Discussion).

### Comparative effectiveness, overall and by age, HbA_1c_ and presence/absence of MLTC

The crude (unadjusted) change in mean HbA_1c_ from baseline to 1 year follow-up was greatest for people prescribed SU (−18.2 mmol/mol [−3.8%] vs either DPP4i −10.1 mmol/mol [−3.1%] or SGLT2i −14.4 mmol/mol [−3.5%]) (ESM Fig. 11).

**Fig. 2:**
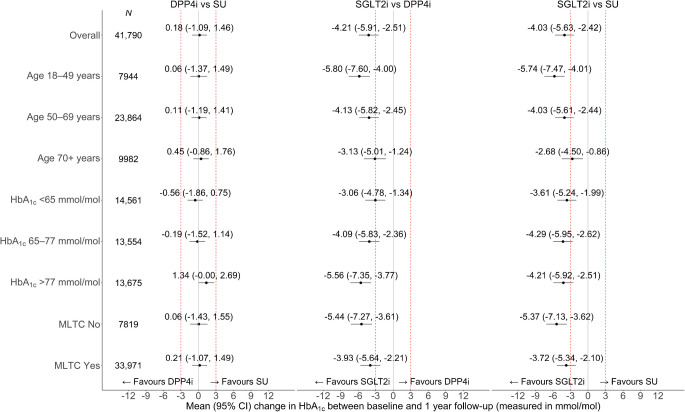
Forest plot showing estimated differences in the change in HbA_1c_ between baseline and 1 year follow-up for DPP4i vs SU, SGLT2i vs DPP4i and SGLT2i vs SU for age, HbA_1c_ and MLTC subgroups. The dashed red lines represent differences of +3 or −3 mmol/mol, which are regarded as of clinical significance

After reducing the risk of confounding with the instrumental variable analysis, we found strong evidence that SGLT2i were more effective than either DPP4i or SU in reducing HbA_1c_ between baseline and 1 year follow-up overall, and by age, baseline HbA_1c_ and the presence of MLTC (Fig. 2). Overall, the difference in mean (95% CI) change was −4.03 mmol/mol (−5.63, −2.42) (−0.4% [−0.5, −0.2]) for SGLT2i vs SU, and −4.21 mmol/mol (−5.91, −2.51) (−0.4% [−0.5, −0.2]) for SGLT2i vs DPP4i. Table 2 and ESM Table 2 report ‘difference in differences’ between subgroups in the change in HbA_1c_ between baseline and 1 year follow-up for each treatment comparison, with HbA_1c_ reported in mmol/mol and in % units, respectively. We also report CIs and *p* values for the null hypothesis of no difference between subgroups.

**Table 2:** Hypothesis testing for differences in the change in HbA_1c_ in mmol/mol between baseline and 1 year follow-up for DPP4i vs SU, SGLT2 vs SU and SGLT2i vs DPP4i comparing age, HbA_1c_ and MLTC subgroups

Group	DP44i vs SU		SGLT2i vs DPP4i		SGLT2i vs SU	
	Difference in differences (95% CI)	*p* value	Difference in differences (95% CI)	*p* value	Difference in differences (95% CI)	*p* value
All participants						
Age						
50–69 vs 18–49 years	0.05 (−0.74, 0.84)	0.903	1.67 (0.82, 2.51)	<0.001	1.71 (0.89, 2.54)	<0.001
70+ vs 18–49 years	0.39 (−0.42, 1.19)	0.346	2.67 (1.65, 3.69)	<0.001	3.06 (2.04, 4.08)	<0.001
70+ vs 50–69 years	0.34 (−0.18, 0.86)	0.204	1.01 (0.26, 1.75)	0.008	1.34 (0.57, 2.12)	0.001
HbA_1c _(mmol/mol)						
65–77 vs <65	0.36 (−0.16, 0.89)	0.170	−1.04 (−1.61, −0.47)	<0.001	−0.67 (−1.29, −0.05)	0.034
>77 vs <65	1.9 (1.23, 2.57)	<0.001	−2.5 (−3.24, −1.76)	<0.001	−0.6 (−1.37, 0.17)	0.124
>77 vs 65–77	1.53 (0.84, 2.23)	<0.001	−1.46 (−2.28, −0.65)	<0.001	0.07 (−0.76, 0.9)	0.869
MLTC						
With vs without	0.14 (−0.76, 1.05)	0.755	1.51 (0.6, 2.42)	0.001	1.65 (0.72, 2.59)	0.001
Participants without MLTC						
Age						
50–69 vs 18–49 years	−0.94 (−2.66, 0.78)	0.285	2.76 (1.07, 4.46)	0.001	1.82 (0.04, 3.61)	0.045
70+ vs 18–49 years	−0.74 (−3.22, 1.75)	0.561	6.17 (2.5, 9.85)	0.001	5.44 (1.66, 9.22)	0.005
70+ vs 50–69 years	0.2 (−2.17, 2.58)	0.867	3.41 (−0.04, 6.86)	0.053	3.61 (0.08, 7.15)	0.045
HbA_1c _(mmol/mol)						
65–77 vs <65 at age 18–49 years	−2.24 (−5.2, 0.72)	0.138	−0.25 (−3.07, 2.56)	0.860	−2.49 (−5.69, 0.7)	0.125
>77 vs <65 at age 18–49 years	1.99 (−1.39, 5.37)	0.248	−3.65 (−7.23, −0.07)	0.046	−1.66 (−5.1, 1.79)	0.345
>77 vs 65–77 at age 18–49 years	4.23 (0.91, 7.56)	0.013	−3.4 (−6.94, 0.14)	0.060	0.84 (−2.54, 4.21)	0.627
65–77 vs <65 at age 50–69 years	1.93 (−0.15, 4)	0.069	−2.51 (−4.5, −0.51)	0.014	−0.58 (−3.02, 1.86)	0.642
>77 vs <65 at age 50–69 years	2.2 (−0.31, 4.71)	0.085	−4.41 (−6.92, −1.9)	0.001	−2.21 (−4.89, 0.47)	0.106
>77 vs 65–77 at 50–69 years	0.27 (−2.24, 2.79)	0.831	−1.9 (−4.48, 0.68)	0.148	−1.63 (−4.44, 1.19)	0.257
65–77 vs <65 at age 70+ years	3.18 (−1.04, 7.39)	0.140	−2.54 (−7.04, 1.96)	0.269	0.64 (−4.64, 5.91)	0.813
>77 vs <65 at age 70+ years	2.81 (−3.12, 8.75)	0.353	4.58 (−6.67, 15.83)	0.425	7.39 (−3.25, 18.04)	0.173
>77 vs 65–77 at age 70+ years	−0.36 (−6.98, 6.26)	0.915	7.12 (−4.2, 18.44)	0.218	6.76 (−4.19, 17.7)	0.226
Participants with MLTC						
Age						
50–69 vs 18–49 years	0.47 (−0.32, 1.26)	0.239	0.84 (−0.06, 1.74)	0.067	1.31 (0.45, 2.18)	0.003
70+ vs 18–49 years	0.75 (−0.04, 1.55)	0.063	1.62 (0.56, 2.68)	0.003	2.37 (1.34, 3.4)	<0.001
70+ vs 50–69 years	0.28 (−0.25, 0.81)	0.299	0.78 (0.04, 1.52)	0.038	1.06 (0.31, 1.81)	0.006
HbA_1c _(mmol/mol)						
65–77 vs <65 at age 18–49 years	0.87 (−0.4, 2.15)	0.179	−0.88 (−2.57, 0.82)	0.310	0 (−1.53, 1.52)	0.996
>77 vs <65 at age 18–49 years	1 (−0.67, 2.67)	0.240	0.02 (−1.98, 2.03)	0.982	1.02 (−0.9, 2.95)	0.297
>77 vs 65–77 at age 18–49 years	0.13 (−1.55, 1.81)	0.883	0.9 (−1.3, 3.1)	0.423	1.03 (−0.91, 2.97)	0.299
65–77 vs <65 at age 50–69 years	0.2 (−0.5, 0.89)	0.582	−0.94 (−1.76, −0.13)	0.023	−0.75 (−1.63, 0.13)	0.094
>77 vs <65 at age 50–69 years	1.72 (0.81, 2.63)	<0.001	−2.54 (−3.54, −1.53)	<0.001	−0.82 (−1.82, 0.18)	0.108
>77 vs 65–77 at age 50–69 years	1.52 (0.55, 2.49)	0.002	−1.59 (−2.71, −0.47)	0.005	−0.07 (−1.18, 1.05)	0.903
65–77 vs <65 at age 70+ years	0.39 (−0.45, 1.23)	0.360	−0.45 (−1.55, 0.65)	0.422	−0.06 (−1.26, 1.14)	0.924
>77 vs <65 at age 70+ years	3.07 (1.98, 4.17)	<0.001	−1.73 (−3.49, 0.03)	0.054	1.34 (−0.43, 3.12)	0.138
>77 vs 65–77 at age 70+ years	2.68 (1.45, 3.91)	<0.001	−1.28 (−3.11, 0.55)	0.170	1.4 (−0.42, 3.22)	0.131

Note: The difference in differences is defined as $\left[{\Delta Y}_{Group2}^{Drug A}-{\Delta Y}_{Group2}^{Drug B}\right]-\left[{\Delta Y}_{Group1}^{Drug A}-{\Delta Y}_{Group1}^{Drug B}\right]$ where $\Delta \text{Y is change in }{\text{HbA}}_{1\text{c}}$. A positive value therefore indicates that the additional change in ${\text{HbA}}_{1\text{c}}$ obtained when using drug A over drug B is greater in group 2 than in group 1

For data presenting HbA_1c_ in % units, see ESM Table 2

The mean improvement in HbA_1c_ following SGLT2i compared with SU was larger for people aged 18–49 years than for those aged 50–69 years (difference in differences: 1.71 mmol/mol [95% CI 0.89, 2.54] [0.2% (0.1, 0.2)]) or aged 70 years or over (3.06 mmol/mol [95% CI 2.04, 4.08] [0.3% (0.2, 0.4)]) (Table 2 and ESM Table 2). For the comparison of SGLT2i with DPP4i, the corresponding mean (95% CI) difference in differences were 1.67 mmol/mol (0.82, 2.51) (0.2% [0.1, 0.2]) for age 50–69 vs age 18–49 years and 2.67 mmol/mol (1.65, 3.69) (0.2% [0.2, 0.3]) for age 70 or over vs age 18–49 years. Although the mean difference (improvement) in HbA_1c_ was greater following SGLT2i than either alternative for people in the groups with lower vs higher HbA_1c_, these differences between strata were small and not of clinical significance.

Mean HbA_1c_ improved to a greater extent following treatment with SGLT2i vs either SU or DPP4i irrespective of the presence of MLTCs. For the comparison of SGLT2i with SU, the mean (95% CI) difference in differences between people with vs without MLTC was 1.65 mmol/mol (0.72, 2.59) (0.2% [0.1, 0.2]). For SGLT2i vs DPP4i the corresponding difference in differences was 1.51 mmol/mol (0.60, 2.42) (0.1% [0.1, 0.2]) and for DPP4i vs SU the difference in differences was small and not of clinical or statistical significance (0.14 mmol/mol [−0.76, 1.05]) (0% [−0.1, 0.1]) (Table 2 and ESM Table 2).

### Comparative effectiveness in individuals without MLTC, according to age and HbA_1c_, alone and combined

**Fig. 3:**
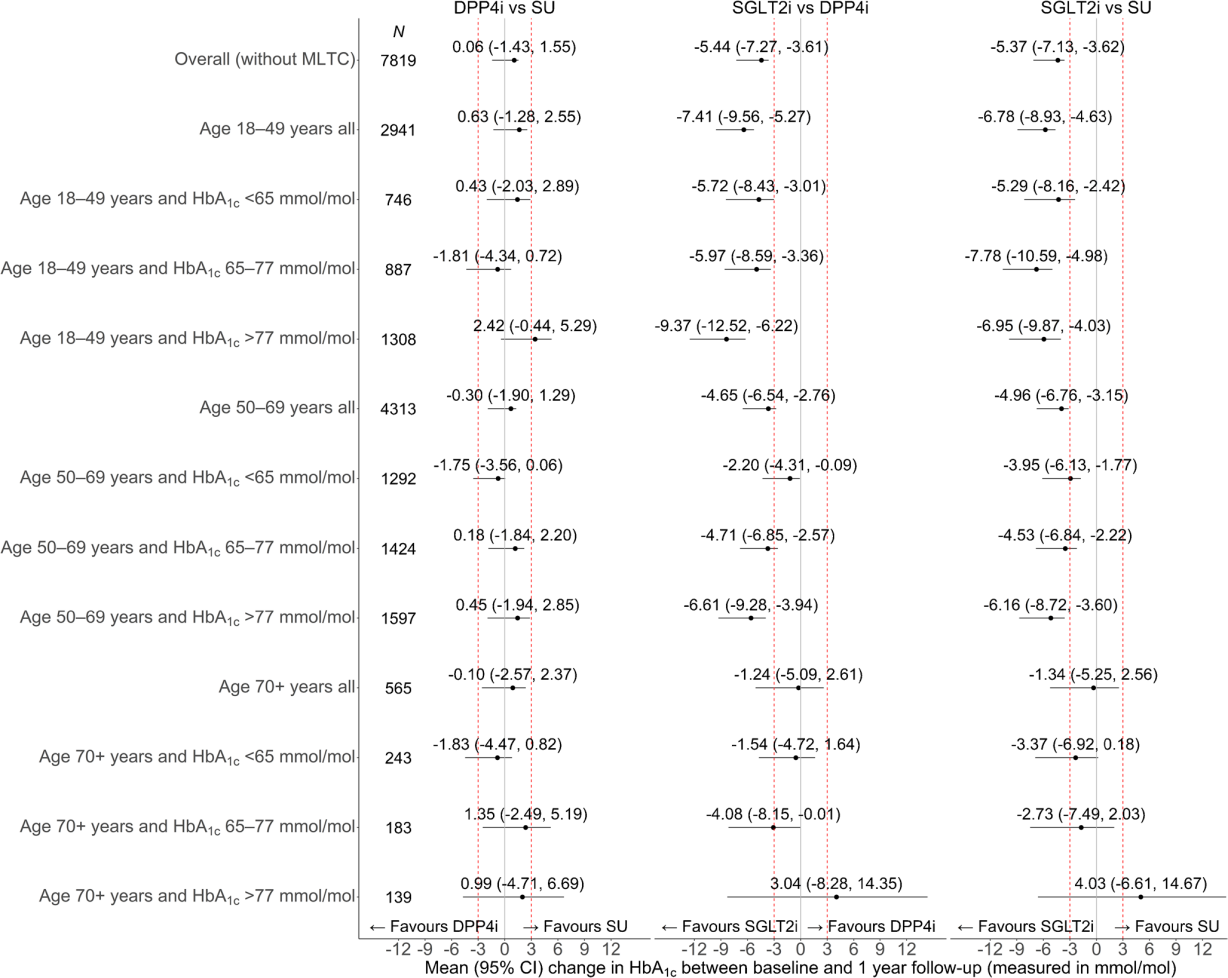
Forest plot showing estimated differences in the change in HbA_1c_ between baseline and 1 year follow-up for DPP4i vs SU, SGLT2i vs DPP4i and SGLT2i vs SU for age and HbA_1c_ subgroups of individuals without MLTC. The dashed red lines represent differences of +3 or −3 mmol/mol, which are regarded as of clinical significance

For people without MLTC, SGLT2i led to improvements in glycaemic control compared with either SU or DPP4i across subgroups defined by age group or level of baseline HbA_1c_ (Fig. 3). For comparisons of SGLT2i with either of the other second-line treatments there was weak evidence of heterogeneity in comparative effectiveness according to age group by level of baseline HbA_1c_ (Table 2 and ESM Table 2). The mean improvement in HbA_1c_ following SGLT2i compared with SU was larger for people aged 18–49 years than for those aged 50–69 years (difference in difference: 1.82 mmol/mol [95% CI 0.04, 3.61] [0.2% (0.0, 0.3)]) or for those aged 70 years or over (5.44 mmol/mol [95% CI 1.66, 9.22]) [0.5% (0.2, 0.8)] (Table 2 and ESM Table 2). For SGLT2i vs DPP4i, the corresponding difference in differences were 2.76 mmol/mol (1.07, 4.46) (0.3% [0.1, 0.4]) and 6.17 mmol/mol (2.50, 9.85) (0.6% [0.2, 0.9]) (Table 2 and ESM Table 2). For participants aged 70 years or over, the mean differences in HbA_1c_ for SGLT2i compared with the other treatments were smaller than for other age groups and were not statistically significantly different from 0 (Fig. 3); this drove statistically significant difference in differences estimates between this age group and the other age groups (Table 2 and ESM Table 2).

### Comparative effectiveness in individuals with MLTC according to age group and baseline HbA_1c_

**Fig. 4:**
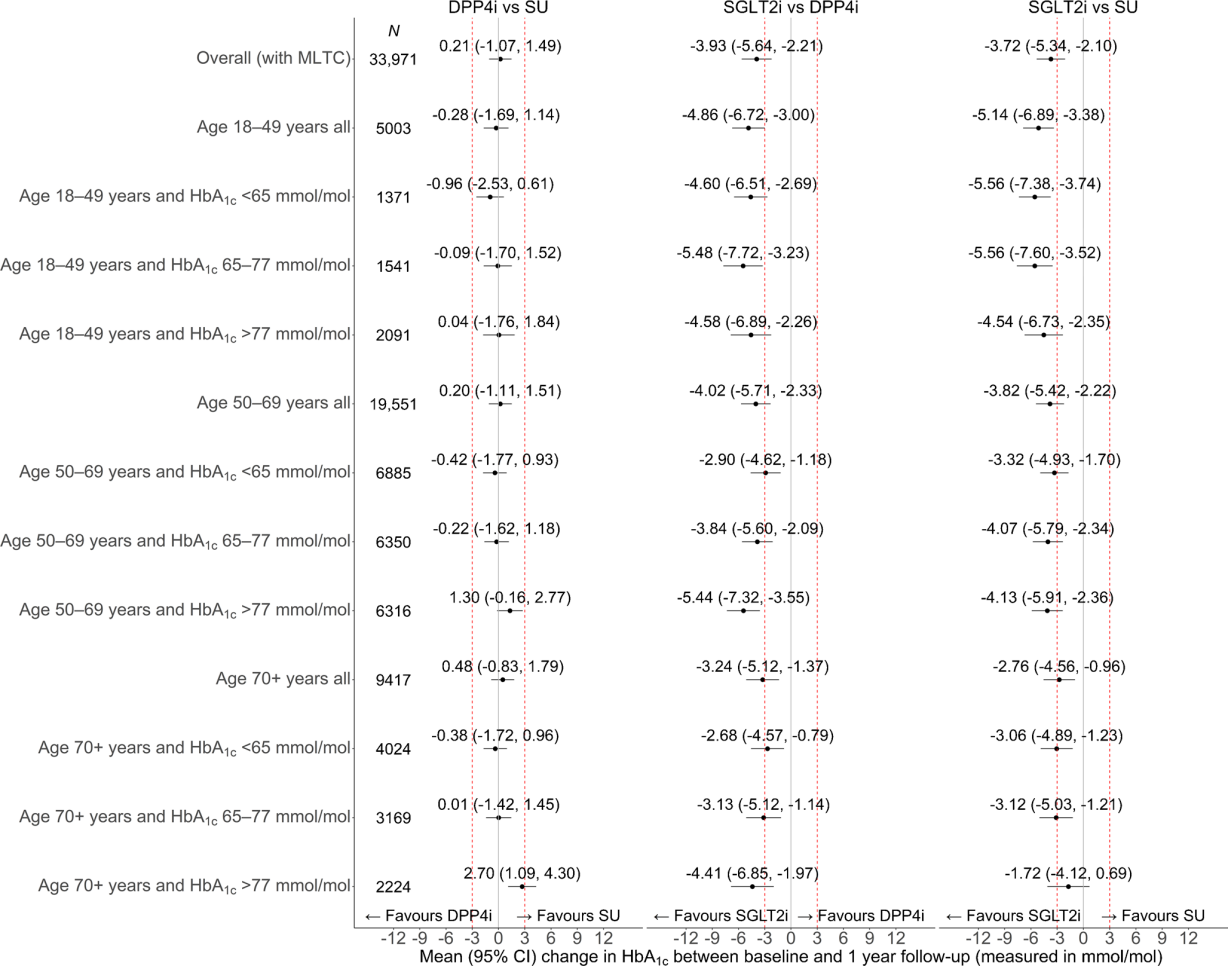
Forest plot showing estimated differences in the change in HbA_1c_ between baseline and 1 year follow-up for DPP4i vs SU, SGLT2i vs DPP4i and SGLT2i vs SU for age and HbA_1c_ subgroups of individuals with MLTC. The dashed red lines represent differences of +3 or −3 mmol/mol, which are regarded as of clinical significance

For people with MLTCs, SGLT2i led to greater reductions in HbA_1c_ than SU or DPP4i overall (Fig. 4). The mean improvement in HbA_1c_ following SGLT2i compared with SU was larger for people aged 18–49 years than for those aged 50–69 years (the mean [95% CI] difference in difference was 1.31 mmol/mol [0.45, 2.18] [0.1% (0.0, 0.2)]) or for those aged 70 years or over (2.37 mmol/mol [1.34, 3.40] [0.2% (0.1, 0.3)]) (Table 2 and ESM Table 2). There was little evidence of heterogeneity by age and baseline HbA_1c_ in the estimates of relative effectiveness (Table 2 and ESM Table 2). For the comparison of SGLT2i vs DPP4i, the corresponding estimated mean (95% CI) difference in differences was 0.84 mmol/mol (−0.06, 1.74) (0.1% [0, 0.2]) and 1.62 mmol/mol (0.56, 2.68) (0.1% [0.1, 0.2]). For DPP4i vs SU, the corresponding difference in differences were small and not of clinical or statistical significance (0.47 mmol/mol [−0.32, 1.26) (0.0% [0.0, 0.1]) and 0.75 mmol/mol (−0.04, 1.55) (0.1% [0.0, 0.1]) (Table 2 and ESM Table 2).

### Comparative effectiveness in individuals with MLTC by age group and baseline HbA_1c_ combined, overall and according to specific MLTCs

**Fig. 5:**
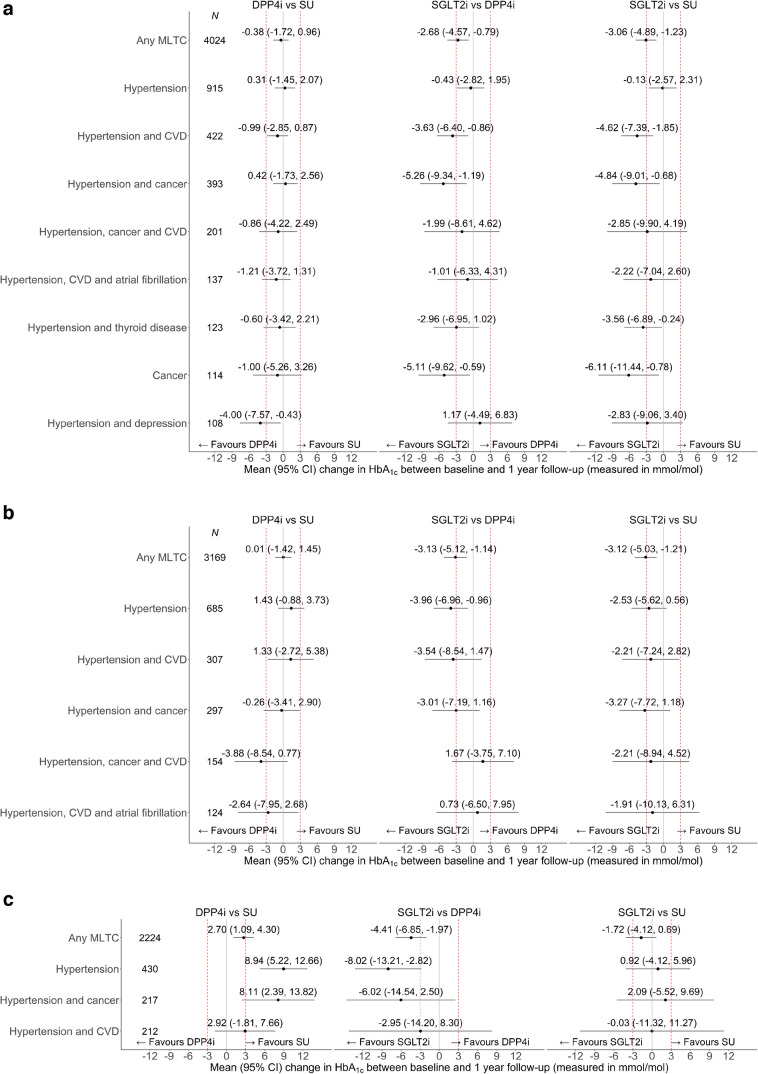
Forest plot showing estimated differences in the change in HbA_1c_ between baseline and 1 year follow-up for DPP4i vs SU, SGLT2i vs DPP4i and SGLT2i vs SU for MLTC profile by HbA_1c_ subgroups of individuals aged 70 years and over: (**a**) HbA_1c_ <65 mmol/mol; (**b**) HbA_1c_ 65–77 mmol/mol; (**c**) HbA_1c_ >77 mmol/mol. The dashed red lines represent differences of +3 or −3 mmol/mol, which are regarded as of clinical significance

There was little evidence of heterogeneity in the estimates of comparative effectiveness according to combinations of age group, baseline HbA_1c_ level and specific MLTCs (Figs 4, 5 and ESM Figs 12, 13). SGLT2i led to improvements in mean HbA_1c_ compared with either SU or DPP4i for subgroups defined by age groups and baseline HbA_1c_ levels combined. While there were variations in the magnitude of the mean reductions in HbA_1c_ following SGLT2i vs either of the alternatives across specific MLTCs, the mean improvements in HbA_1c_ were in favour of SGLT2i for almost all of the underlying MLTCs (Figs 4, 5 and ESM Figs 12, 13).

## Discussion

We found that second-line treatment with SGLT2i was more effective than SU or DPP4i in reducing HbA_1c_ between baseline and 1 year across subgroups defined by age, baseline HbA_1c_ and MLTCs. For most subgroups we found that SGLT2i remained more effective in improving glycaemic management compared with SU or DPP4i. We found some evidence that for people without MLTC the comparative effectiveness of SGLT2i in reducing HbA_1c_ was attenuated by increasing age. However, among people living with MLTCs, SGLT2i were more effective than DPP4i or SU in reducing HbA_1c_ across subgroups defined by age and HbA_1c_, and across most MLTCs. The estimates of comparative effectiveness for specific MLTCs within age and HbA_1c_ subgroups were highly uncertain and should be regarded as hypothesis generating.

National guidelines [4] and international guidance [39] for treatment of people with type 2 diabetes do not provide nuanced recommendations as to which patients benefit most from a given treatment since most of the relevant RCTs only assess average treatment effects in selected trial populations [7–11, 40–43]. Genetic data are not widely available, and so precision medicine for type 2 diabetes relies on demographic characteristics, biomarkers and information about MLTC [4]. However, for older people living with MLTCs the evidence to support treatment choice and HbA_1c_ targets is limited [4]. Our finding that the comparative effectiveness of SGLT2i in reducing HbA_1c_ may be attenuated by age is consistent with a network meta-analysis of RCTs [12] and a previous causal analysis that used a similar instrumental approach and data to the PERMIT study [18].

A possible mechanism for attenuation in the effectiveness of SGLT2i vs DPP4i according to HbA_1c_ in older people is that because SGLT2i act through inhibiting the active reabsorption of glucose in the proximal tubule, impaired renal function is associated with smaller improvement in glycaemic control [44, 45]. The TriMaster study found that in individuals with relatively low eGFR (60–90 ml/min per 1.73 m^2^), which is associated with increasing age, a DPP4i (sitagliptin) led to greater improvements in HbA_1c_ compared with an SGLT2i (canagliflozin) [15]. SGLT2i can lead to decreases in BP, leading to a risk of hypotension and falls, and there is a lack of evidence supporting their use in older adults with frailty [46].

The PERMIT study [20] considers all three common second-line oral glucose-lowering treatments for a general population of people with type 2 diabetes, including older people, those with MLTCs and those with relatively high baseline HbA_1c_. The PERMIT study previously found that compared with either of the alternative second-line treatments, SGLT2i were effective in reducing mean HbA_1c_, BMI, SBP, heart failure hospitalisation and eGFR decline [20] but has not previously considered subgroups (other than previous CVD) alone or in combination. Our finding that second-line treatment with SGLT2i may lead to improved glycaemic management compared with DPP4i or SU for subgroups at higher baseline HbA_1c_ levels and with MLTCs builds on previous RCT evidence [47, 48]. When we examined baseline HbA_1c_ and age combined, rather than separately, we did not find strong evidence of effect modification by HbA_1c_ within the respective age subgroups. Nor did we find strong evidence of differences in comparative effectiveness according to specific MLTC profiles. However, the sample size for the profiles according to age group, baseline HbA_1c_ level and individual MLTC profiles were small and this is reflected in the wide 95% CI around the comparative effectiveness estimates. The uncertainty estimates do not allow for multiple testing and so the comparative effectiveness estimates for the patient profiles defined by specific MLTCs combined with age and HbA_1c_ strata should be viewed as generating hypotheses for future research.

Previous studies used CPRD data [14, 49, 50] to inform personalised approaches to type 2 diabetes. Dennis et al. [14] used forward selection to develop a treatment selection model to predict HbA_1c_ outcomes for after SGLT2i or DPP4i therapy. The study found that clinical characteristics such as age, baseline HbA_1c_, BMI, eGFR and alanine aminotransferase were associated with differential levels of improvement in HbA_1c_. Cardoso et al. [49] used Bayesian causal forests to develop an individualised treatment selection algorithm for SGLT2i and glucagon-like peptide-1 receptor agonists (GLP1-RAs). Venkatasubramaniam et al. [50] reported that a regression-based model performed substantially better than a causal forest approach for identifying strata with a clinically important observed treatment benefit for SGLT2i vs DPP4i. These studies, which used advanced methods to explore treatment heterogeneity, are still at risk of bias in the estimates of comparative effectiveness due to unmeasured confounding, albeit it is reassuring that their findings were successfully replicated in RCTs.

In our study, people prescribed SGLT2i had fewer LTCs and were likely to be healthier according to unmeasured baseline characteristics including overall health, diet, exercise and lifestyle. We reduced the risk of unobserved confounding by using an instrumental variable approach that avoided the ‘no unmeasured confounding’ assumption [23]. Our study pre-specified a limited number of comparisons for hypothesis testing that were informed by the extant literature (according to age, HbA_1c_ and prevalent MLTC status, alone and in combination). We also aimed to generate hypotheses for many subgroups (~100) for the individual MLTC stratified by age and baseline HbA_1c_.

The study has several strengths. First, we used a large primary and secondary care-linked dataset [22] that included a multiethnic population with a range of baseline HbA_1c_, ages and MLTCs, including those usually excluded from RCTs. While we did not report results stratified by biological sex, the large representative nature of the sample allows the results to be generalised to similar patient groups. Second, we took a target trial emulation approach [24] combined with an instrumental variable analysis to reduce the risk of confounding when assessing comparative effectiveness [23]. Third, we considered mutually exclusive groups so that the evidence generated relates directly to specific patient subgroups defined not just by single risk factors (age, baseline HbA_1c_, presence of MLTC) but also the most prevalent combinations of these risk factors.

This paper has some limitations. We did not consider cardiorenal outcomes. Previous research has reported that SGLT2i improve cardiorenal outcomes, although these studies did not compare directly with either SU or DPP4i [7, 9, 11, 17]. It was not possible to consider long-term cardiorenal complications due to their low prevalence among the subgroups; this led to non-convergence of models. Further research should explore treatment effect heterogeneity and personalisation across a wide range of potential treatment options (including GLP1-RAs) for all important type 2 diabetes-related outcomes. Previous evidence has suggested the potential for effect modification according to renal function, but in our sample only 2795 (6.7%) of the individuals had eGFR <60 ml/min per 1.73 m^2^. The study’s findings are dependent on the validity of the instrumental variable assumptions, which we assess. Generalised instrumental inequalities could be used as complementary checks of the underlying assumptions [51]. Another potential limitation is that our study was not powered to consider adverse events such as hypoglycaemia or diabetic ketoacidosis; however, particularly for diabetic ketoacidosis, our study would have been underpowered to detect clinically meaningful differences in the incidence of this rare outcome across treatments and clinical subgroups of interest [52]. We did not validate the treatment effect estimates in external data. Validating causal effect estimates is challenging given that only the outcomes of one treatment at a particular timepoint can be observed. A more promising avenue for future research would be to develop decision support tools populated with relevant clinical information from the electronic health record and embed these tools within clinical practice software systems. Future work could then assess the effectiveness of a decision support tool through a cluster randomised trial [53].

### Conclusion

In conclusion, we found that initiating second-line treatment with SGLT2i is more effective than SU or DPP4i in reducing HbA_1c_ between baseline and 1 year follow-up across subgroups of people with type 2 diabetes according to age, baseline HbA_1c_ and the presence or absence of MLTCs. We have provided evidence that uses information available in routine practice on demographic characteristics, biomarkers and MLTC that can improve precision in the choice of glucose-lowering treatment for type 2 diabetes mellitus.

## Supplementary Information

Below is the link to the electronic supplementary material.

ESM (PDF 3380 KB)

## Data Availability

CPRD data of the form used in this paper are available by application to the CPRD Independent Scientific Advisory Committee (https://www.cprd.com/data-access).

## Abbreviations

CCG: Clinical commissioning group
CKD: Chronic kidney disease
COPD: Chronic obstructive pulmonary disease
CPRD: Clinical Practice Research Datalink
DBP: Diastolic BP
DPP4i: Dipeptidyl peptidase-4 inhibitors
GLP1-RA: Glucagon-like peptide-1 receptor agonist
HES: Hospital Episode Statistics
LASSO: Least absolute shrinkage and selection operator
LTC: Long-term condition
MLTC: Multiple long-term condition
NICE: National Institute for Health and Care Excellence
SBP: Systolic BP
SGLT2i: Sodium–glucose cotransporter-2 inhibitors
2SRI: Two-stage residual inclusion
SU: Sulfonylureas
TTP: Tendency to prescribe

## Authors’ relationships and activities

KK has acted as a consultant for, has been a speaker for, or has received grants for investigator-initiated studies from Astra Zeneca, Bayer, Novo Nordisk, Sanofi-Aventis, Servier, Lilly, Merck Sharp & Dohme, Boehringer Ingelheim, Oramed Pharmaceuticals, Pfizer, Roche, Daiichi-Sankyo, Applied Therapeutics, Embecta and Nestle Health Science. AIA has received research income from Novo Nordisk. The authors declare that there are no other relationships or activities that might bias, or be perceived to bias, their work.
