# Supplementary material for: Comparative effectiveness of alternative second-line oral glucose-lowering therapies for type 2 diabetes: a precision medicine approach applied to routine data
Source: Diabetologia. 2025 May 31;68(9):1908–23. doi: 10.1007/s00125-025-06447-x (PMC12361298; doi:10.1007/s00125-025-06447-x)
Supplement: Supplementary file 1 — ESM (PDF 3380 KB) [file 125_2025_6447_MOESM1_ESM.pdf]

Electronic supplementary material (ESM)

Comparative effectiveness of alternative second-line oral glucose-lowering therapies in people with type 2 diabetes: a precision medicine approach applied to routine data

Contents

ESM Methods 3

Study design and participants 3

Covariates 3

Statistical analysis 3

ESM Tables 7

ESM Table 1: Number and percentage of patients with a given number of comorbidities* 8

ESM Table 2: Hypothesis testing for differences in the change in HbA_{1c} (%) between baseline and 1-year follow-up for (i) DPP4i compared to SU, (ii) SGLT2 compared to SU, and (iii) SGLT2i compared to DPP4i comparing age, HbA_{1c} (%) and MLTC subgroups 9

ESM Figures 11

ESM Fig. 1: Stacked bar chart illustrating the variation in second-line glucose lowering therapy prescribed among people included in the study at the clinical commissioning group (CCG)-level in England, 2014-2020 12

ESM Fig. 2: Study population inclusion flow diagram 13

ESM Fig. 3: Variation in prescribing of SU, DPP4i and SGLT2i by age, HbA_{1c} and MLTC subgroups in 2019 and 2020 15

ESM Fig. 4: Variation in prescribing of SU, DPP4i and SGLT2i by age, HbA_{1c} and MLTC subgroups in 2015 to 2018 16

ESM Fig. 5: Variation in prescribing of SU, DPP4i and SGLT2i by age and HbA_{1c} subgroups for those without MLTC 17

ESM Fig. 6: Variation in prescribing of SU, DPP4i and SGLT2i by age and HbA_{1c} subgroups for those with MLTC 18

ESM Fig. 7: Variation in prescribing of SU, DPP4i and SGLT2i for MLTC profile by HbA_{1c} subgroups for those aged 18 to 49 years 19

ESM Fig. 8: Variation in prescribing of SU, DPP4i and SGLT2i for MLTC profile by HbA_{1c} subgroups for those aged 50 to 69 years 20

ESM Fig. 9: Variation in prescribing of SU, DPP4i and SGLT2i for MLTC profile by HbA_{1c} subgroups for those aged 70 years and over. 23

ESM Fig. 10: Mean level of rescaled baseline covariates according to the level of the instrumental variable 26

ESM Fig. 11: Crude (unadjusted) mean HbA_{1c} and change in mean HbA_{1c} from baseline to 0.5, 1, and 2-years follow-up for those who initiated second-line oral glucose-lowering treatment with one of sulfonylureas (SU), dipeptidyl peptidase-4 inhibitors (DPP4i), and sodium-glucose co-transporter 2 inhibitors (SGLT2i) all added to metformin monotherapy 28

ESM Fig. 12: Forest plot showing differences in the change in HbA_{1c} between baseline and 1-year follow-up for (i) DPP4i compared to SU, (ii) SGLT2 compared to SU, and (iii) SGLT2i compared to DPP4i for MLTC profile by HbA_{1c} subgroups for those aged 18 to 49 years. 29

ESM Fig. 13: Forest plot showing differences in the change in HbA_{1c} between baseline and 1-year follow-up for (i) DPP4i compared to SU, (ii) SGLT2 compared to SU, and (iii) SGLT2i compared to DPP4i for MLTC profile by HbA_{1c} subgroups for those aged 50 to 69 years. 32

References 35

ESM Methods

Study design and participants

Individuals aged 18 years and over, registered with a GP in England who intensified treatment from first- to second-line oral glucose lowering therapies between 1 January 2015 to 31 December 2020 with a first-ever prescription of SU, DPP4i or SGLT2i, added to metformin were included. Those eligible had at least one prescription for metformin monotherapy within 60 days prior to the first prescription for second-line treatment, to ensure their use of metformin monotherapy was continuous prior to intensification. We excluded women with a record of pregnancy within 12 months prior to second-line treatment initiation and people whose last recorded eGFR was less than 30mL/min/1.73m^2, since prescribing guidelines recommend different treatments for these groups. We also excluded people whose GP practices had not consented to the required linkage of HES data. We followed precedent research in excluding those who were not prescribed metformin on the same day, or within 60 days after initiating second-line treatment,[1] as it is unlikely that their treatment with metformin continued.

Covariates

We defined patient sociodemographic characteristics (age, sex, ethnicity, index of multiple deprivation (IMD)), time since T2DM diagnosis, year of second-line glucose lowering therapy initiation, NHS region (East of England, London, Midlands, North East and Yorkshire, North West, South East, and South West),[2] number of patients registered with the participants GP practice, smoking and alcohol status, relevant co-prescriptions (renin-angiotensin system inhibitors (RASi) or statins) issued within 60 days prior to baseline, hospitalisation (any) in the previous year, and LTCs recorded prior to baseline. We also defined $HbA_{1c,}$ SBP, diastolic blood pressure (DBP), eGFR, and BMI[3] using the most recent measures recorded in primary care. For HbA_{1c} we only considered the most recent measure within 180 days prior to baseline, and for SBP, DBP, and eGFR, we considered the most recent measure within 540 days prior to baseline. Any values recorded prior to these time windows were considered out-dated and were not used in defining baseline characteristics.

Statistical analysis

Estimation approach:

Suppose that Treatment is determined by a multinomial model as follows:

$$\log\left(\frac{\Pr(D=k|X_D)}{\Pr(D=0|X_D)}\right) = \alpha_k X_D + \alpha_{U_k} u_k + \alpha_{Z_k} Z_k$$

where X_D is a vector of covariates that influence treatment choice, u_k is an unobserved confounder influencing the choice of treatment *k* and Z_k and instrumental variable that is independent of u_k. The probability of a particular treatment¸ *k*, being chosen is given by:

$$\Pr(D = k|X_D) = \frac{\exp(\alpha_k X_D + \alpha_{U_k} u_k + \alpha_{Z_k} Z_k)}{\sum_{j=0}^{2} \exp\left(\alpha_j X_D + \alpha_{U_j} u_j + \alpha_{Z_j} Z_j\right)}$$

and the observed outcome, $D = \{D_{SU}, D_{DPP4i}, D_{SGLT2i}\}$, is the outcome with the highest probability of occurrence.

The outcome, Y, is determined as follows:

$$Y^* = X_Y \mathrm{B} + \tau_{DPP4i}(1 + M_{Y,DPP4i}\delta_{DPP4i}) * D_{DPP4i} + \tau_{SGLT2i}(1 + M_{Y,SGLT2i}\delta_{SGLT2i}) * D_{SGLT2i} + U + \epsilon$$

Where X_Y is vector of covariates that influence the outcome, M_k is a vector of effect modifiers for treatment *k*, with the strength of modification captured by the parameters δ_1 and δ_2, and U captures unobserved confounders which can be separated into those that influence each treatment (U_{DPP4i} and U_{SGLT2i}). We assume that a strong valid instrument is available for each treatment.

Instrumental variable assumptions:

A valid instrument must (i) predict the treatment prescribed, which can be formally assessed,[4] (ii) be independent of covariates that predict the outcomes of interest, and (iii) have an effect on the outcomes only through the treatment received. Large imbalances in measured covariates across levels of the TTPs would raise concerns about the second and third IV assumptions. Support for these assumptions is offered by precedent work[5] and in analyses presented in the supplement (**ESM Fig. 1 & 2**). We assessed the extent to which observed prognostic covariates differed across levels of the instrument (see **ESM Fig. 2**). A lack of correlation between the instrument and prognostic covariates, increases the plausibility that unobserved confounders are uncorrelated with the instrument

Confounder selection:

The challenges we consider here are that (a) we are uncertain which variables to include in X_D, X_Y, $M_{Y,DPP4i}$ and $M_{Y,SGLT2i}$ and (b) that there is endogeneity when $U = \{U_{DPP4i}, U_{SGLT2i}\}$ influences the choice of treatment D where U is defined relative to its value for SU ($U_{DPP4i} = u_{DPP4i} - U_{SU}$). We proceed as follows:

Step 1: Apply LASSO to a model relating the outcome to the full set of covariates to the sample of individuals that did not receive either of the treatments of interest (DPP4i and SGLT2i) i.e. those on SU. Store the selected variables, V_Y.

Step 2: Apply LASSO to a logit model relating the receipt of DPP4i to the full set of covariates, and forcing the inclusion of the instruments Z_{DPP4i} and Z_{SGLT2i}. Store the selected variables, V_{DPP4i} (excluding the IVs).

Step 3: Apply LASSO to a logit model relating the receipt of SGLT2i to the full set of covariates, and forcing the inclusion of the instruments Z_{DPP4i} and Z_{SGLT2i}. Store the selected variables, V_{SGLT2i} (excluding the IVs).

Step 4: Take the union of the selected variables $V = V_Y \cup V_{DPP4i} \cup V_{SGLT2i}$. Then estimate a multinomial logit model for the treatment (*D*) as a function of V and the instruments Z_{DPP4i} and Z_{SGLT2i} and predict the generalized residual for each treatment level ($Resid_{SU}$, $Resid_{DPP4i}$, $Resid_{SGLT2i}$).

Step 5: Then to identify potential effect modifiers, run LASSO on the outcome model forcing the inclusion of V, $Resid_{DPP4i}$, $Resid_{SGLT2i}$, and identifiers for the characteristics/morbidity profiles, and allowing LASSO to select interactions between the treatment identifiers (D_{DPP4i} and D_{SGLT2i}) and the set of potential modifiers M to capture effect modifiers and for the full sample.

Step 6: Finally estimate an outcome model that includes the profiles, V, $Resid_{DPP4i}$, $Resid_{SGLT2i}$ and the profiles and selected M*D interactions. This model is then used to estimate individual level and subgroup level effect estimates using recycled predictions (StataCorp 2021[6]; Basu and Rathouz, 2005[7]). Inference is carried out by bootstrapping. Due to computational complexity, we do not include the LASSO steps in the bootstrap, although standard errors do not appear to be sensitive to this based on preliminary estimates using 50 bootstraps.

We reported differences between the comparison groups according to absolute change in outcomes between baseline and follow-up for HbA_{1c}. We reported results overall and according to patient profiles (subgroups). To recognise statistical uncertainty in the estimates of treatment effects, the data were bootstrapped 500 times, stratified by profile and treatment group.

To mitigate bias effectively, our combination of Post-Double Selection (PDS) for variable selection and Two-Stage Residual Inclusion (2SRI) requires several key assumptions to hold. PDS assumes that the true model is sparse, meaning only a small subset of the potential confounders are actually relevant PDS should be robust to moderate model specification mistakes (Belloni et al 2016) in the two selection steps, while 2SRI guards against the possibility that some confounders are unobserved, provided the instruments meet the requirements

outlined above, and both the treatment assignment (first stage) and the outcome (second stage) models are correctly specified and the parameters consistently estimated.

ESM Tables

ESM Table 1: Number and percentage of patients with a given number of comorbidities*

Number of Comorbidities	Number of individuals	%
0	7819	18.71
1	13,250	31.71
2	10,039	24.02
3	5633	13.48
4	2882	6.9
5	1334	3.19
6	526	1.26
7	208	0.5
8	65	0.16
9	27	0.06
10	6	0.01
12	1	<0.01

*CVD counted as 1 comorbidity

ESM Table 2: Hypothesis testing for differences in the change in HbA_{1c} (%) between baseline and 1-year follow-up for (i) DPP4i compared to SU, (ii) SGLT2 compared to SU, and (iii) SGLT2i compared to DPP4i comparing age, HbA_{1c} (%) and MLTC subgroups

	**DP44i vs SU**		**SGLT2i vs DPP4i**		**SGLT2i vs SU**	
**All patients**	**Difference in differences (95% CI)**	**P-value**	**Difference in differences (95% CI)**	**P-value**	**Difference in differences (95% CI)**	**P-value**
Age: 50-69 vs 18-49	0.0 (-0.1, 0.1)	0.903	0.2 (0.1, 0.2)	<0.001	0.2 (0.1, 0.2)	<0.001
Age: 70+ vs 18-49	0.0 (0.0, 0.1)	0.346	0.2 (0.2, 0.3)	<0.001	0.3 (0.2, 0.4)	<0.001
Age: 70+ vs 50-69	0.0 (0.0, 0.1)	0.204	0.1 (0, 0.2)	0.008	0.1 (0.1, 0.2)	0.001
HbA1c: 8.3 to 9.2% vs <8.3%	0.0 (0.0, 0.1)	0.170	-0.1 (-0.1, 0)	<0.001	-0.1 (-0.1, 0)	0.034
HbA1c: >9.2% vs <8.3%	0.2 (0.1, 0.2)	<0.001	-0.2 (-0.3, -0.2)	<0.001	-0.1 (-0.1, 0)	0.124
HbA1c: >9.2% vs 8.3 to 9.2%	0.1 (0.1, 0.2)	<0.001	-0.1 (-0.2, -0.1)	<0.001	0 (-0.1, 0.1)	0.869
MLTC: with vs without	0.0 (-0.1, 0.1)	0.755	0.1 (0.1, 0.2)	0.001	0.2 (0.1, 0.2)	0.001
For those without MLTC:						
Age: 50-69 vs 18-49	-0.1 (-0.2, 0.1)	0.285	0.3 (0.1, 0.4)	0.001	0.2 (0, 0.3)	0.045
Age: 70+ vs 18-49	-0.1 (-0.3, 0.2)	0.561	0.6 (0.2, 0.9)	0.001	0.5 (0.2, 0.8)	0.005
Age: 70+ vs 50-69	0.0 (-0.2, 0.2)	0.867	0.3 (0, 0.6)	0.053	0.3 (0, 0.7)	0.045
HbA1c: 8.3 to 9.2% vs <8.3% with age 18-49	-0.2 (-0.5, 0.1)	0.138	0 (-0.3, 0.2)	0.860	-0.2 (-0.5, 0.1)	0.125
HbA1c: >9.2% vs <8.3% with age 18-49	0.2 (-0.1, 0.5)	0.248	-0.3 (-0.7, 0)	0.046	-0.2 (-0.5, 0.2)	0.345
HbA1c: >9.2% vs 8.3 to 9.2% with age 18-49	0.4 (0.1, 0.7)	0.013	-0.3 (-0.6, 0)	0.060	0.1 (-0.2, 0.4)	0.627
HbA1c: 8.3 to 9.2% vs <8.3% with age 50-69	0.2 (0, 0.4)	0.069	-0.2 (-0.4, 0)	0.014	-0.1 (-0.3, 0.2)	0.642
HbA1c: >9.2% vs <8.3% with age 50-69	0.2 (0, 0.4)	0.085	-0.4 (-0.6, -0.2)	0.001	-0.2 (-0.4, 0)	0.106
HbA1c: >9.2% vs 8.3 to 9.2% mmol/mol with age 50-69	0 (-0.2, 0.3)	0.831	-0.2 (-0.4, 0.1)	0.148	-0.1 (-0.4, 0.1)	0.257
HbA1c: 8.3 to 9.2% vs <8.3% with age 70+	0.3 (-0.1, 0.7)	0.140	-0.2 (-0.6, 0.2)	0.269	0.1 (-0.4, 0.5)	0.813
HbA1c: >9.2% vs <8.3%with age 70+	0.3 (-0.3, 0.8)	0.353	0.4 (-0.6, 1.4)	0.425	0.7 (-0.3, 1.7)	0.173
HbA1c: >9.2% vs 8.3 to 9.2% with age 70+	0.0 (-0.6, 0.6)	0.915	0.7 (-0.4, 1.7)	0.218	0.6 (-0.4, 1.6)	0.226
For those with MLTC:						

	DP44i vs SU		SGLT2i vs DPP4i		SGLT2i vs SU	
**All patients**	**Difference in differences (95% CI)**	**P-value**	**Difference in differences (95% CI)**	**P-value**	**Difference in differences (95% CI)**	**P-value**
Age: 50-69 vs 18-49	0.0 (0.0, 0.1)	0.239	0.1 (0.0, 0.2)	0.067	0.1 (0.0, 0.2)	0.003
Age: 70+ vs 18-49	0.1 (0.0, 0.1)	0.063	0.1 (0.1, 0.2)	0.003	0.2 (0.1, 0.3)	<0.001
Age: 70+ vs 50-69	0.0 (0.0, 0.1)	0.299	0.1 (0.0, 0.1)	0.038	0.1 (0.0, 0.2)	0.006
HbA1c: 8.3 to 9.2% vs <8.3% with age 18-49	0.1 (0.0, 0.2)	0.179	-0.1 (-0.2, 0.1)	0.310	0.0 (-0.1, 0.1)	0.996
HbA1c: >9.2% vs <8.3% with age 18-49	0.1 (-0.1, 0.2)	0.240	0 (-0.2, 0.2)	0.982	0.1 (-0.1, 0.3)	0.297
HbA1c: >9.2% vs 8.3 to 9.2% with age 18-49	0.0 (-0.1, 0.2)	0.883	0.1 (-0.1, 0.3)	0.423	0.1 (-0.1, 0.3)	0.299
HbA1c: 8.3 to 9.2% vs <8.3% with age 50-69	0.0 (0.0, 0.1)	0.582	-0.1 (-0.2, 0)	0.023	-0.1 (-0.1, 0.0)	0.094
HbA1c: >9.2% vs <8.3% with age 50-69	0.2 (0.1, 0.2)	<0.001	-0.2 (-0.3, -0.1)	<0.001	-0.1 (-0.2, 0.0)	0.108
HbA1c: >9.2% vs 8.3 to 9.2% with age 50-69	0.1 (0.1, 0.2)	0.002	-0.1 (-0.2, 0)	0.005	0.0 (-0.1, 0.1)	0.903
HbA1c: 8.3 to 9.2% vs <8.3% with age 70+	0.0 (0.0, 0.1)	0.360	0 (-0.1, 0.1)	0.422	0.0 (-0.1, 0.1)	0.924
HbA1c: >9.2% vs <8.3% with age 70+	0.3 (0.2, 0.4)	<0.001	-0.2 (-0.3, 0)	0.054	0.1 (0.0, 0.3)	0.138
HbA1c: >9.2% vs 8.3 to 9.2% with age 70+	0.2 (0.1, 0.4)	<0.001	-0.1 (-0.3, 0.1)	0.170	0.1 (0.0, 0.3)	0.131

ESM Figures

ESM Fig. 1: Stacked bar chart illustrating the variation in second-line glucose lowering therapy prescribed among people included in the study at the clinical commissioning group (CCG)-level in England, 2014-2020

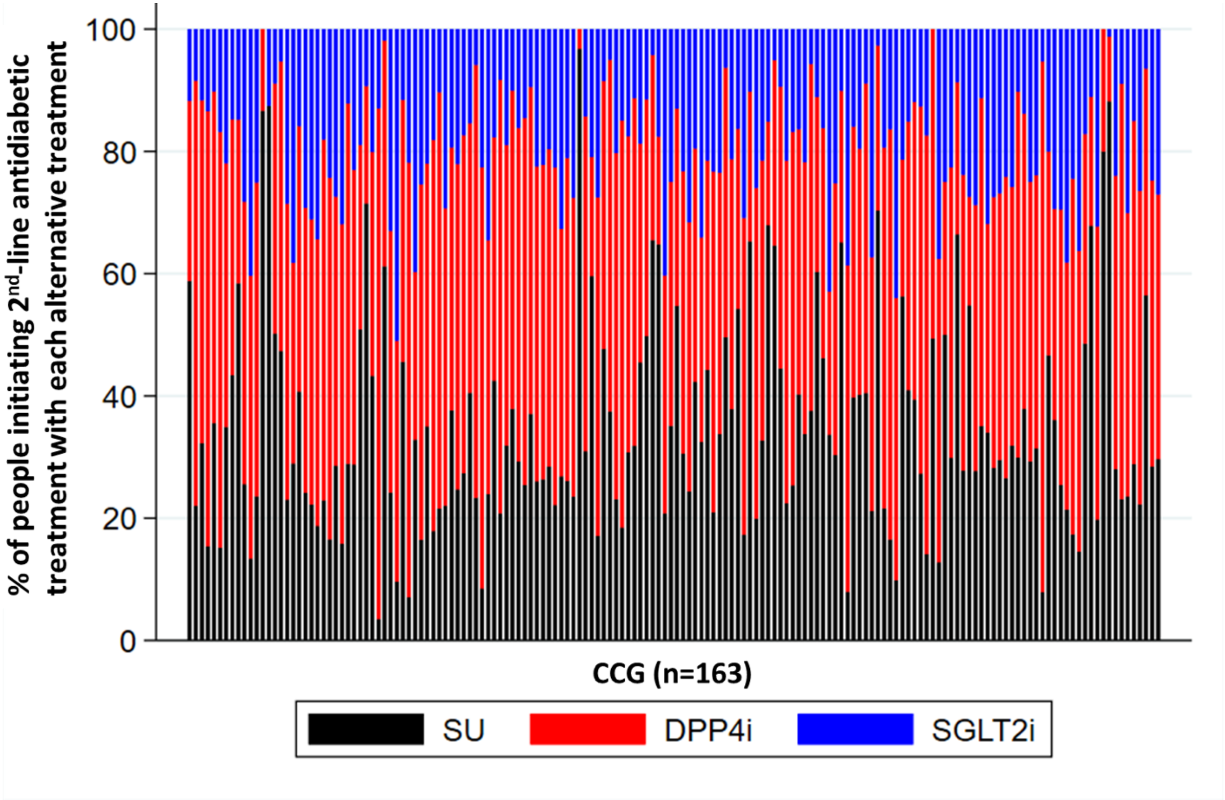

ESM Fig. 2: Study population inclusion flow diagram

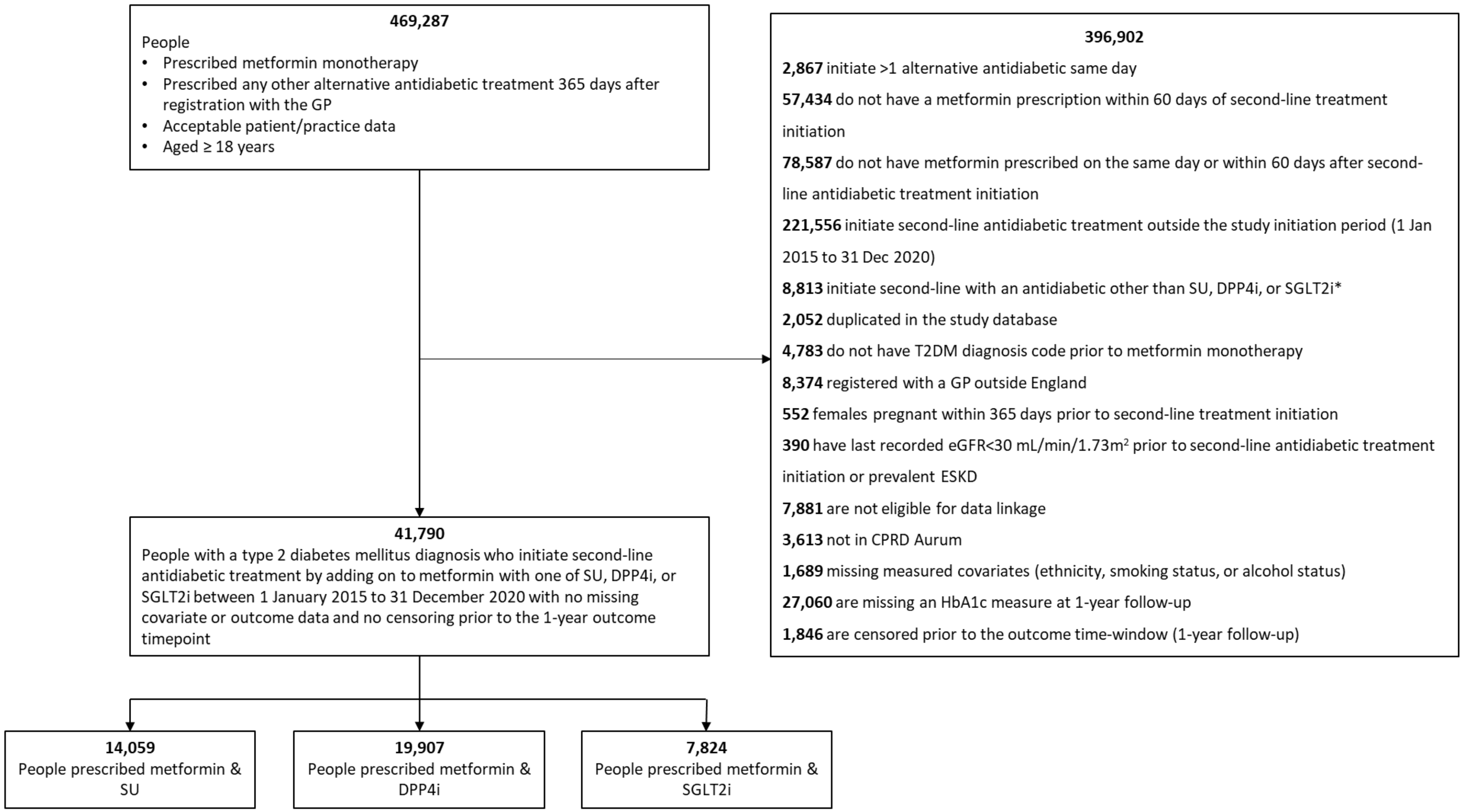

Note that an individual may have met more than one of the criteria for exclusion.

ESM Fig. 3: Variation in prescribing of SU, DPP4i and SGLT2i by age, HbA_{1c} and MLTC subgroups in 2019 and 2020

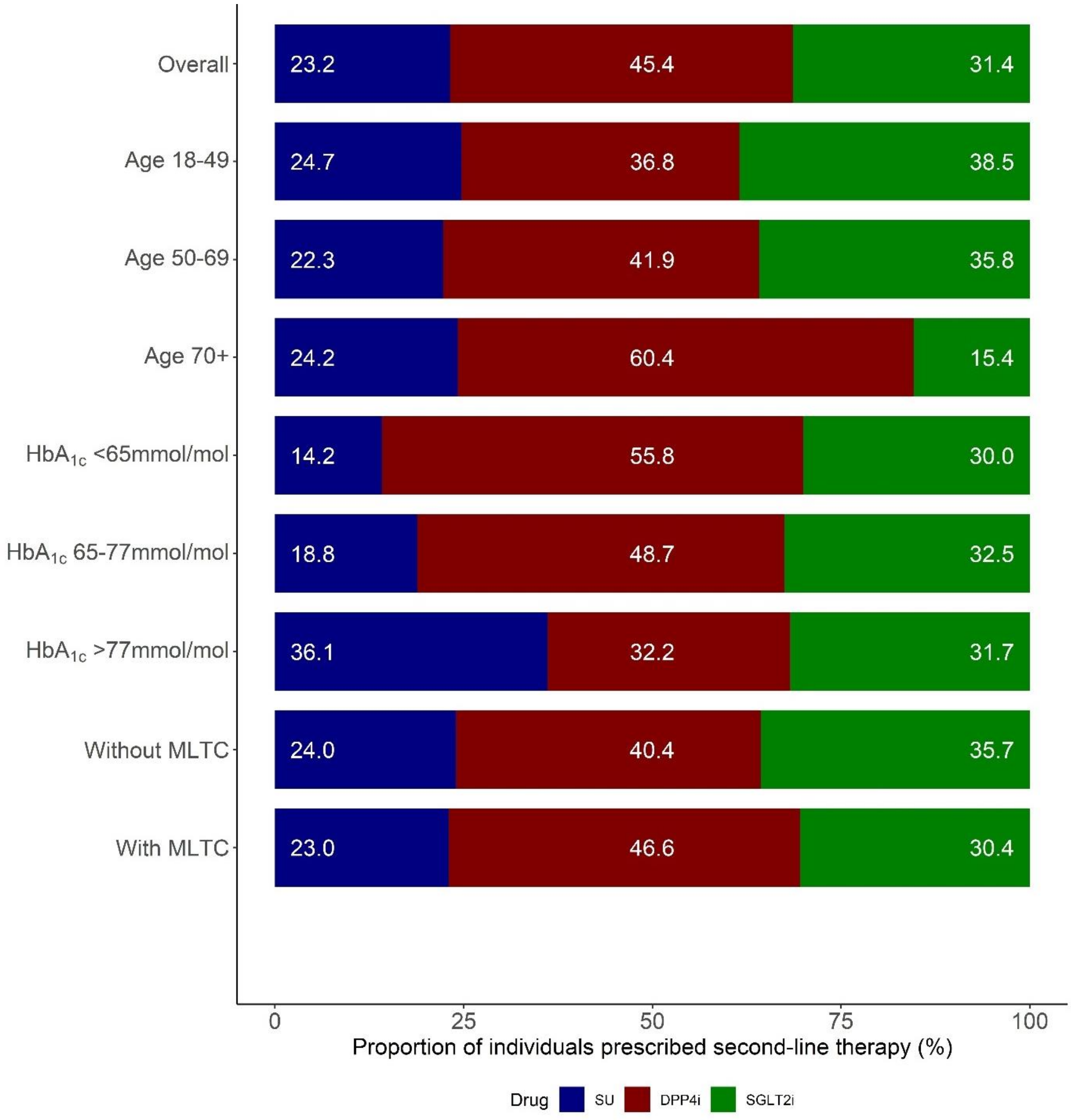

ESM Fig. 4: Variation in prescribing of SU, DPP4i and SGLT2i by age, HbA_{1c} and MLTC subgroups in 2015 to 2018

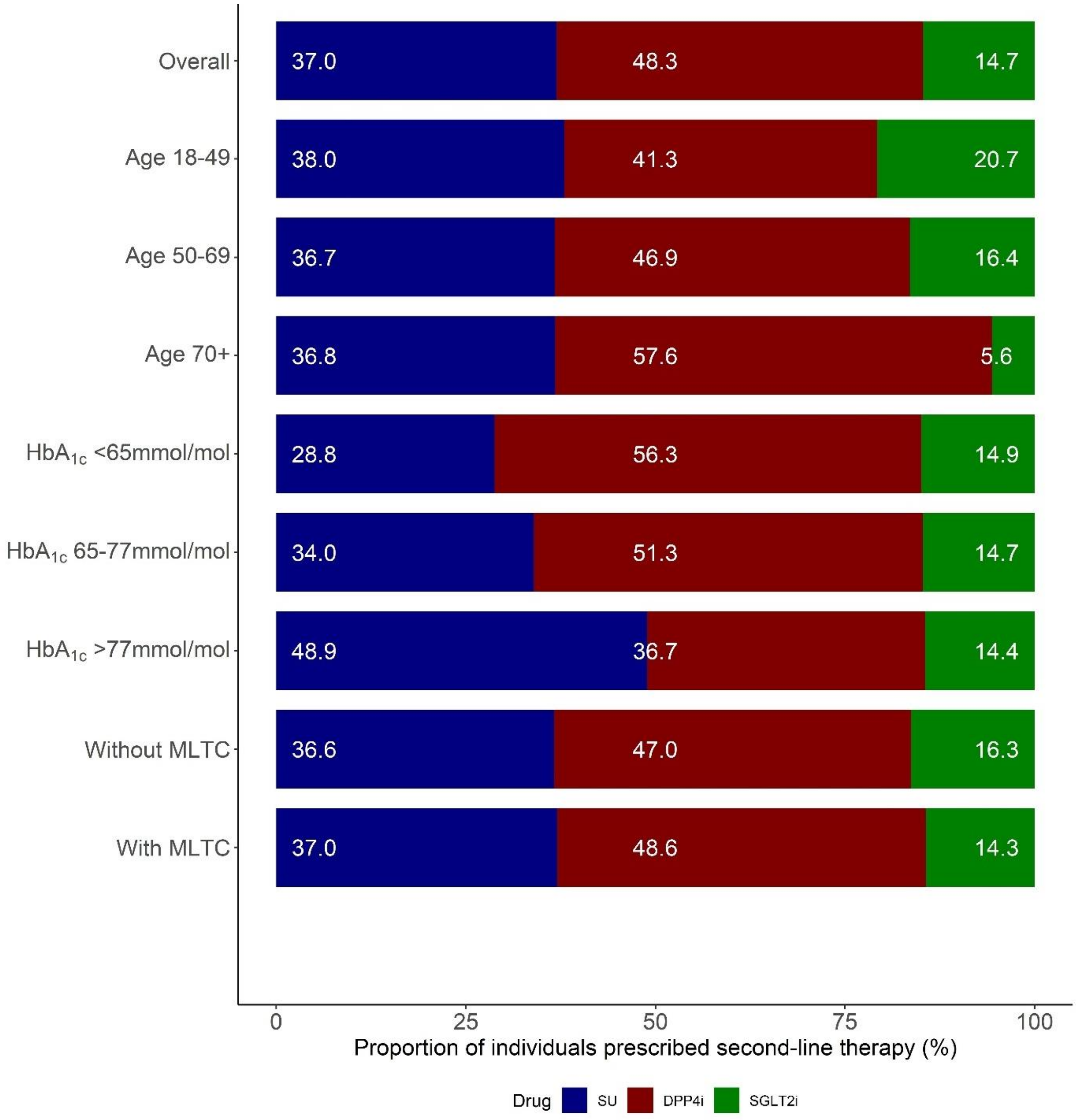

ESM Fig. 5: Variation in prescribing of SU, DPP4i and SGLT2i by age and HbA_{1c} subgroups for those without MLTC

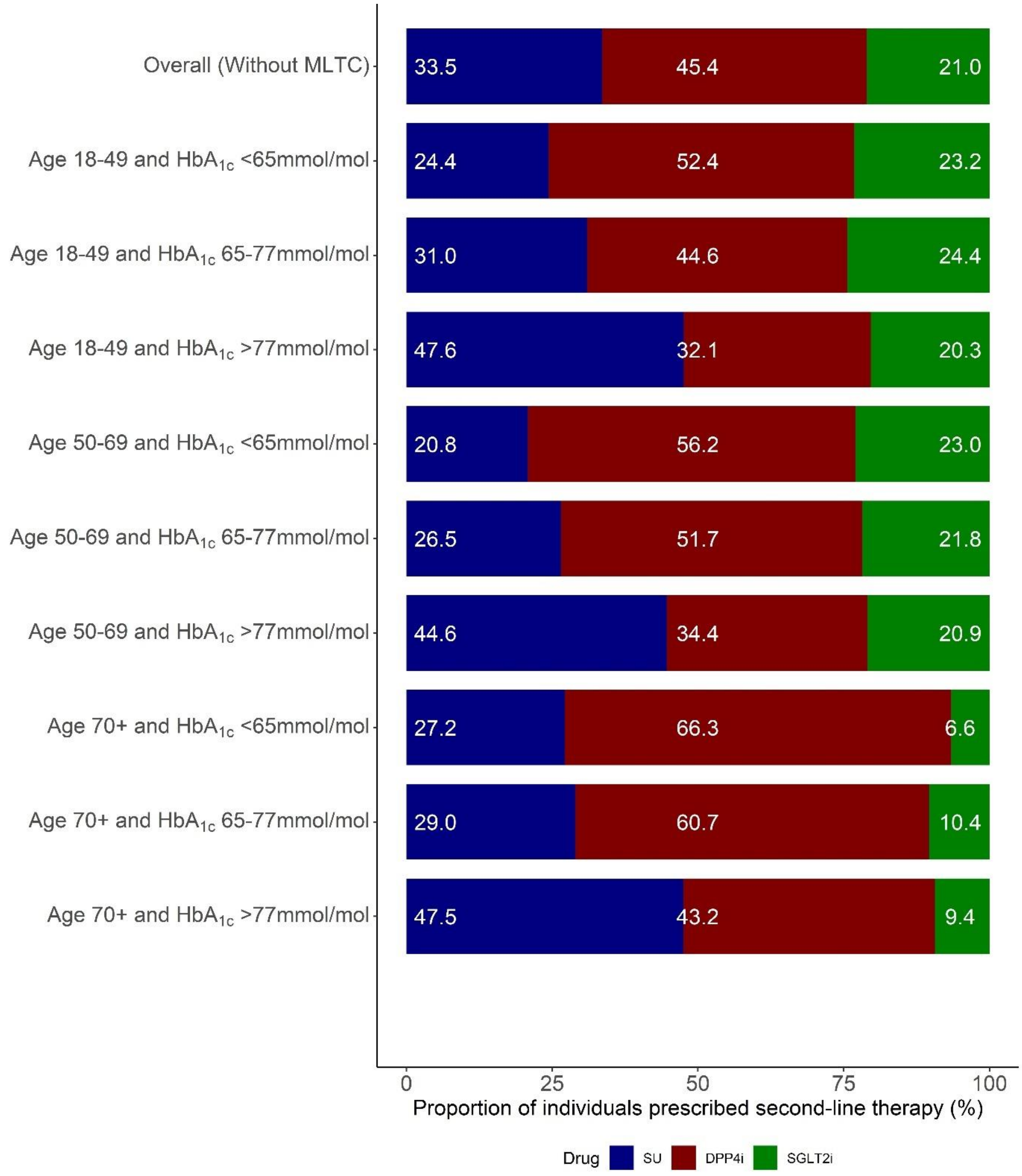

ESM Fig. 6: Variation in prescribing of SU, DPP4i and SGLT2i by age and HbA_{1c} subgroups for those with MLTC

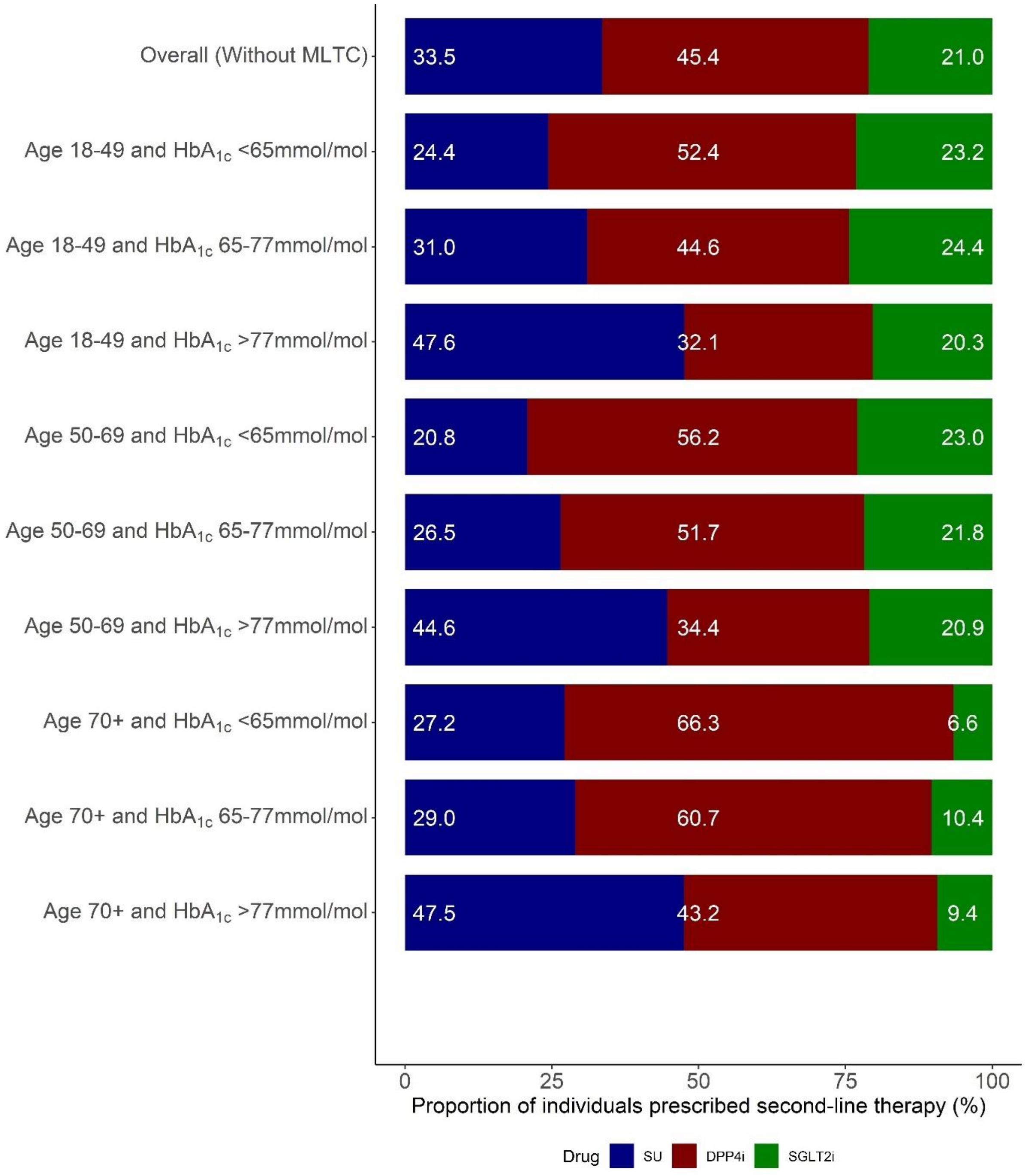

ESM Fig. 7: Variation in prescribing of SU, DPP4i and SGLT2i for MLTC profile by HbA_{1c} subgroups for those aged 18 to 49 years.

(a)

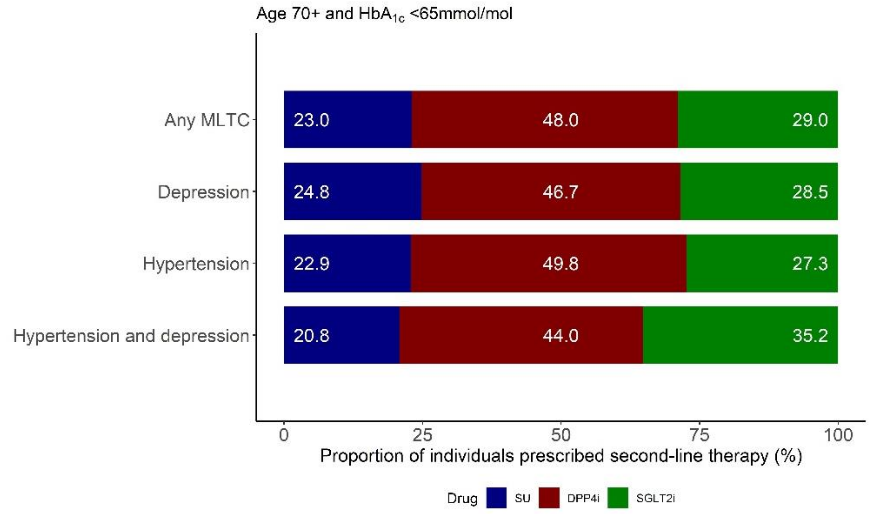

(b)

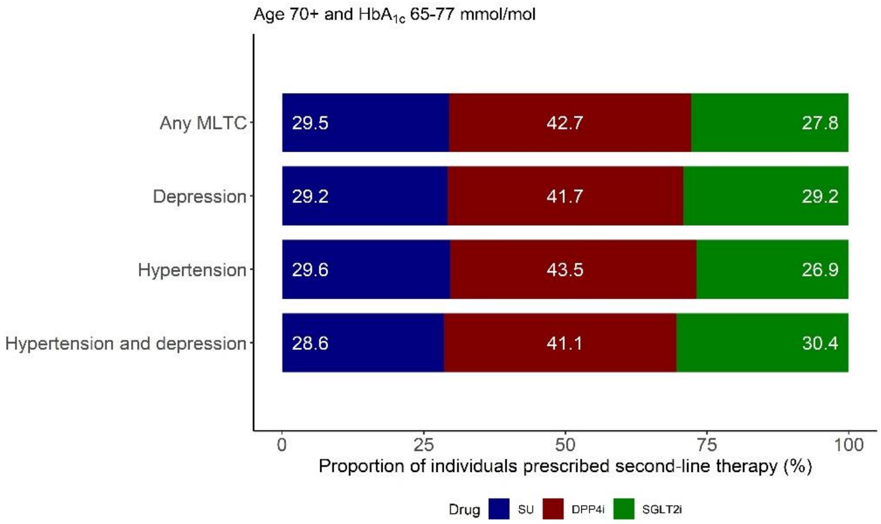

(c)

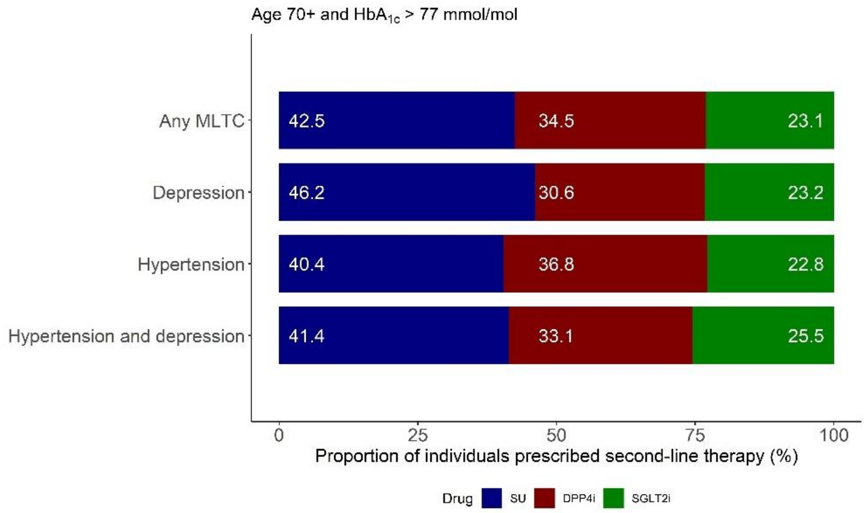

ESM Fig. 8: Variation in prescribing of SU, DPP4i and SGLT2i for MLTC profile by HbA_{1c} subgroups for those aged 50 to 69 years.

(a)

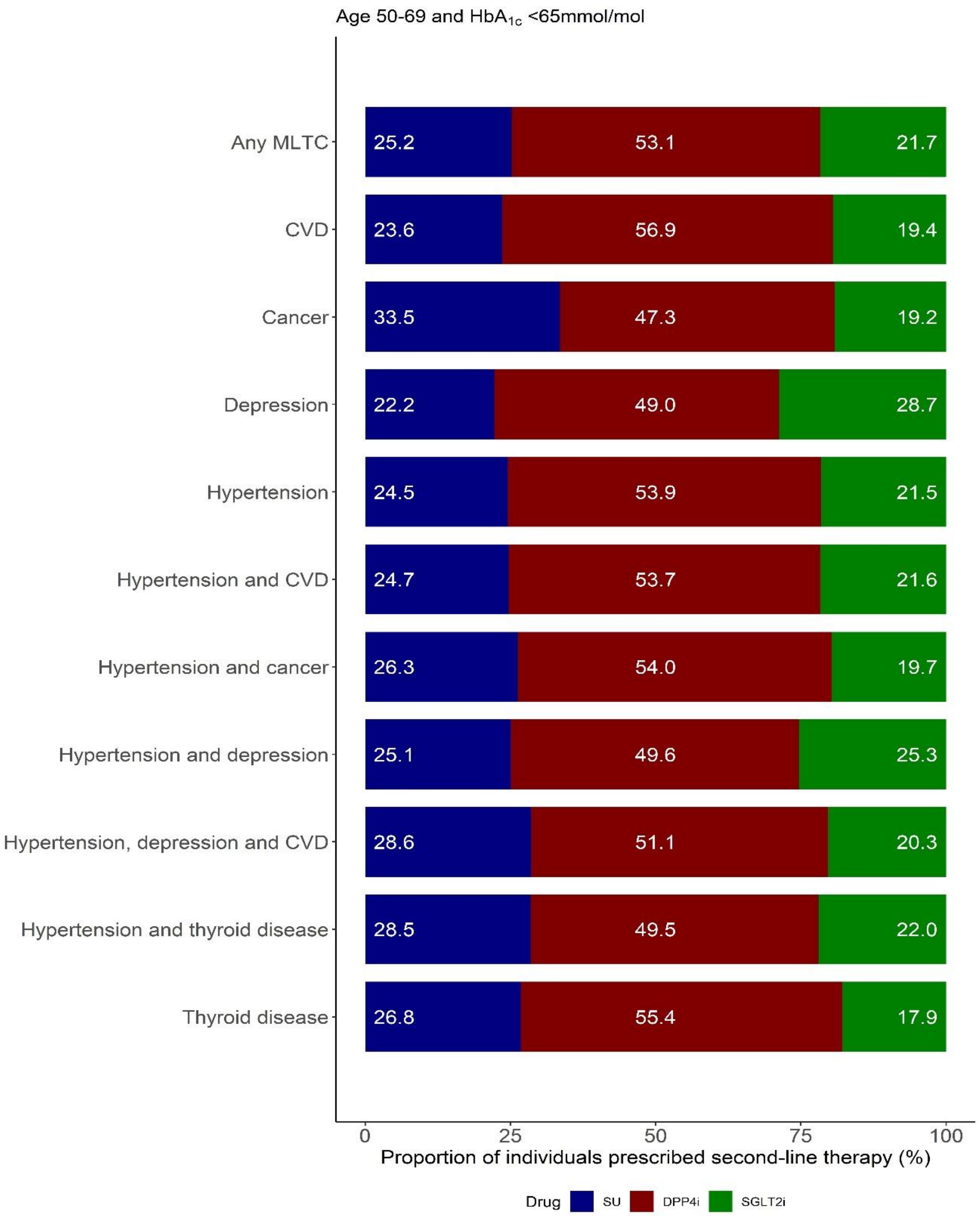

(b)

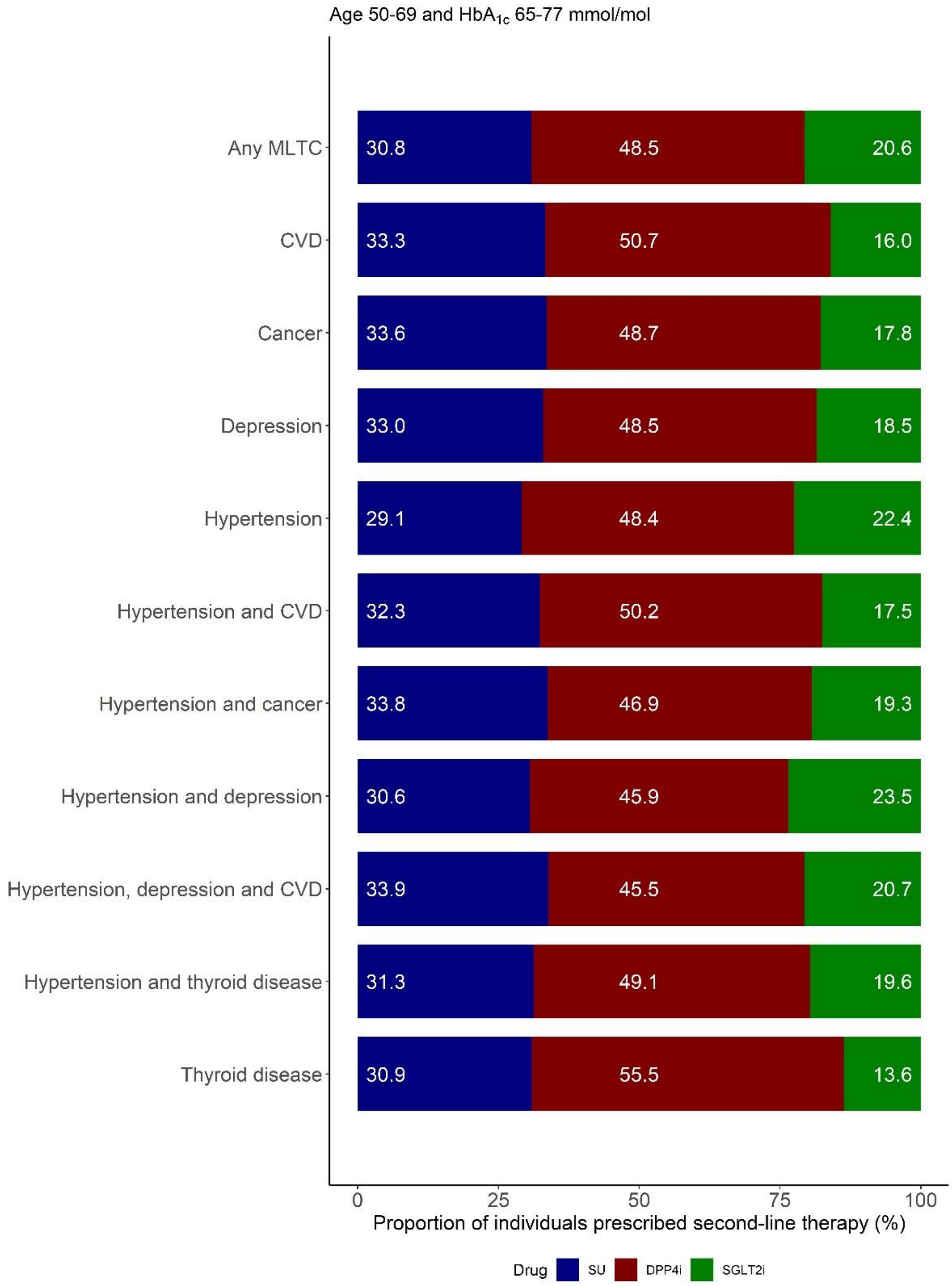

Age 50-69 and HbA1c 65-77 mmol/mol
Any MLTC
30.8
48.5
20.6
CVD
33.3
50.7
16.0
Cancer
33.6
48.7
17.8
Depression
33.0
48.5
18.5
Hypertension
29.1
48.4
22.4
Hypertension and CVD
32.3
50.2
17.5
Hypertension and cancer
33.8
46.9
19.3
Hypertension and depression
30.6
45.9
23.5
Hypertension, depression and CVD
33.9
45.5
20.7
Hypertension and thyroid disease
31.3
49.1
19.6
Thyroid disease
30.9
55.5
13.6
0
25
50
75
100
Proportion of individuals prescribed second-line therapy (%)
Drug
SU
DPP4i
SGLT2i

(c)

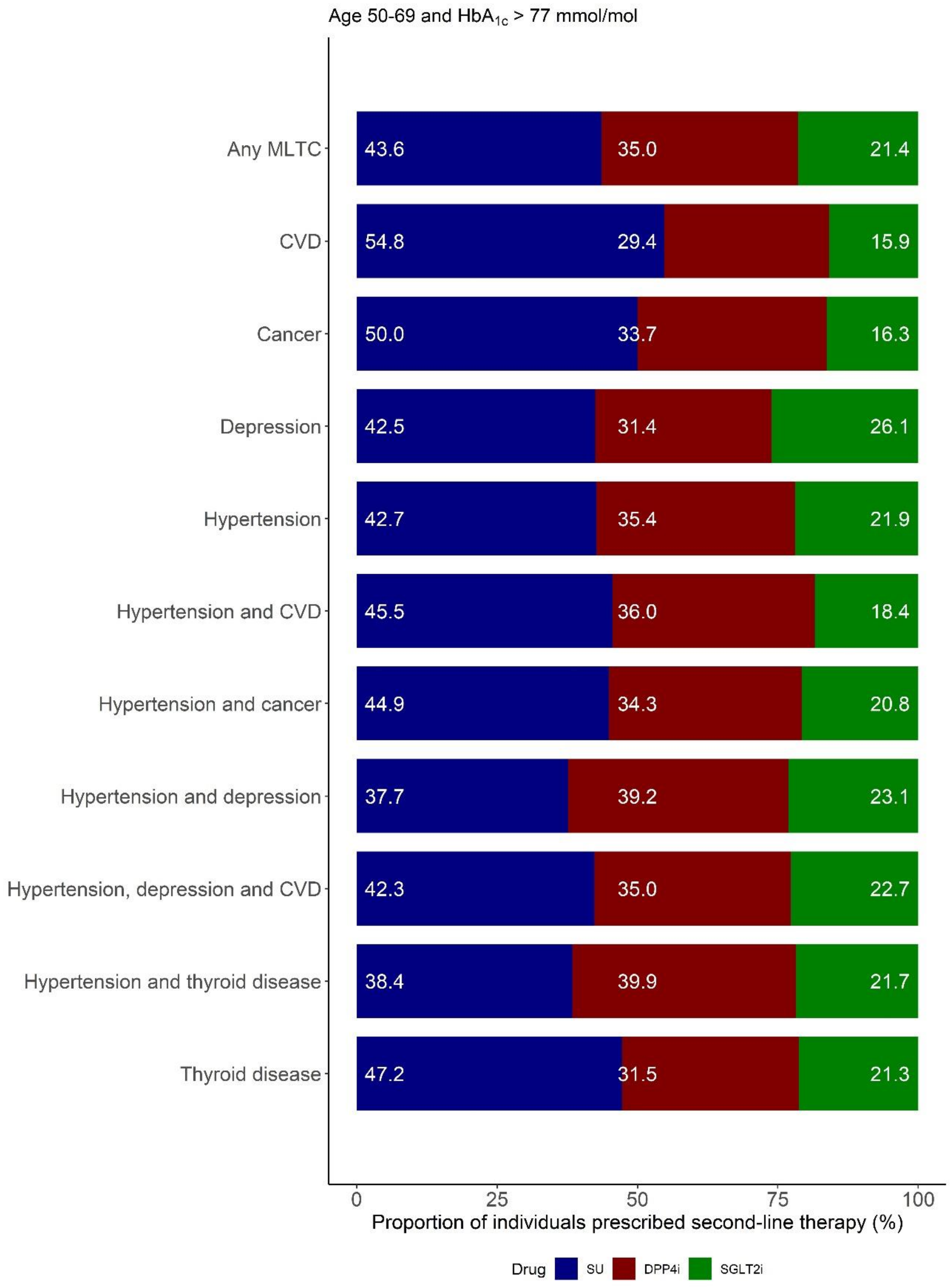

Age 50-69 and HbA_{1c} > 77 mmol/mol
Any MLTC
43.6
35.0
21.4
CVD
54.8
29.4
15.9
Cancer
50.0
33.7
16.3
Depression
42.5
31.4
26.1
Hypertension
42.7
35.4
21.9
Hypertension and CVD
45.5
36.0
18.4
Hypertension and cancer
44.9
34.3
20.8
Hypertension and depression
37.7
39.2
23.1
Hypertension, depression and CVD
42.3
35.0
22.7
Hypertension and thyroid disease
38.4
39.9
21.7
Thyroid disease
47.2
31.5
21.3
0
25
50
75
100
Proportion of individuals prescribed second-line therapy (%)
Drug
SU
DPP4i
SGLT2i

ESM Fig. 9: Variation in prescribing of SU, DPP4i and SGLT2i for MLTC profile by HbA_{1c} subgroups for those aged 70 years and over.

(a)

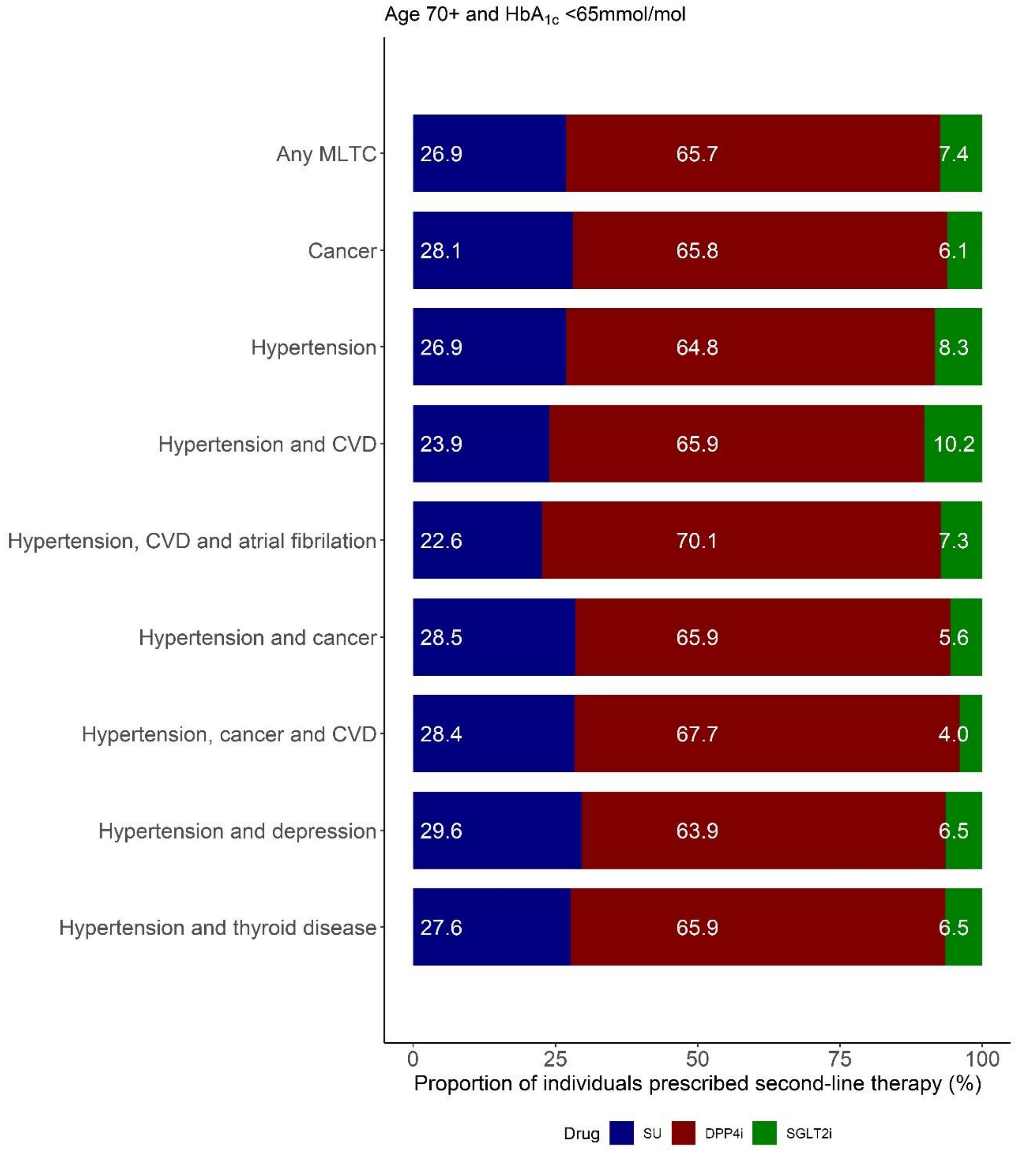

(b)

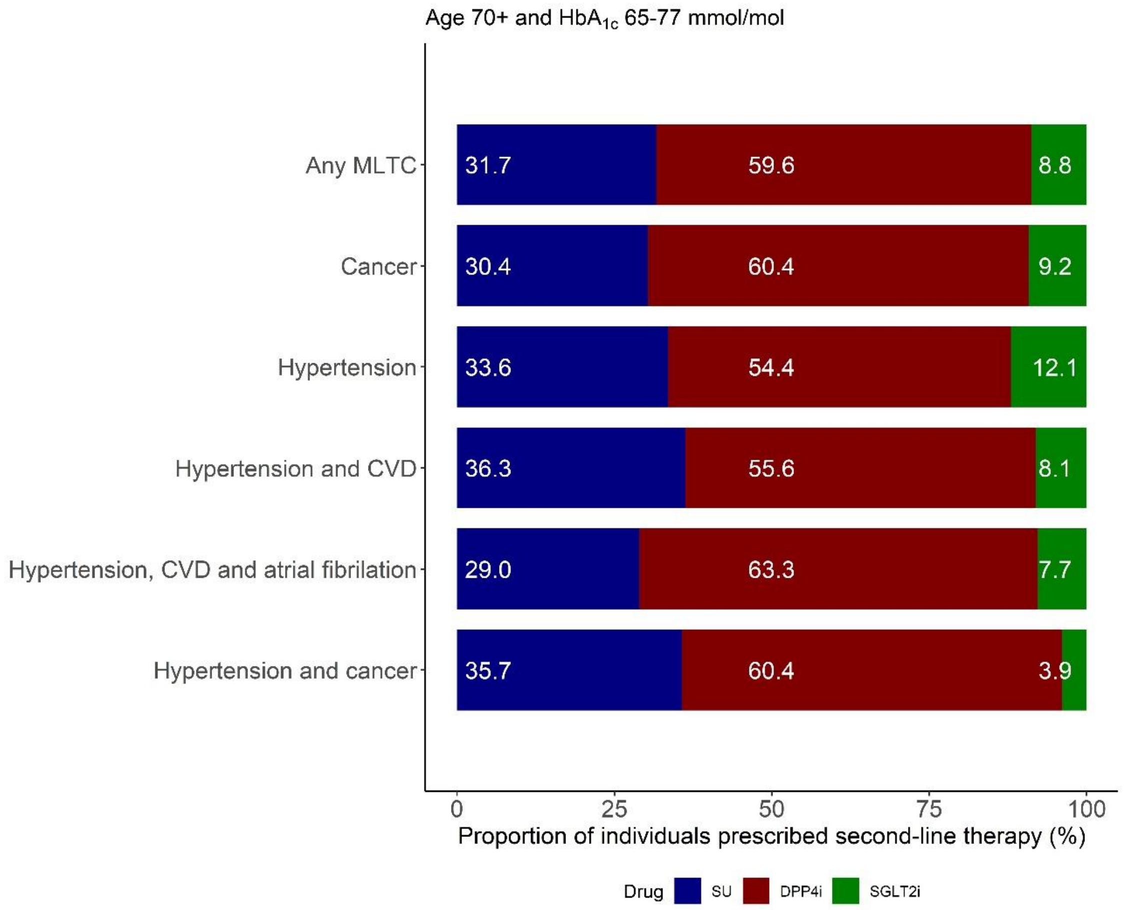

Age 70+ and HbA1c 65-77 mmol/mol
Any MLTC
Cancer
Hypertension
Hypertension and CVD
Hypertension, CVD and atrial fibrilation
Hypertension and cancer
31.7
59.6
8.8
30.4
60.4
9.2
33.6
54.4
12.1
36.3
55.6
8.1
29.0
63.3
7.7
35.7
60.4
3.9
0
25
50
75
100
Proportion of individuals prescribed second-line therapy (%)
Drug
SU
DPP4i
SGLT2i

(c)

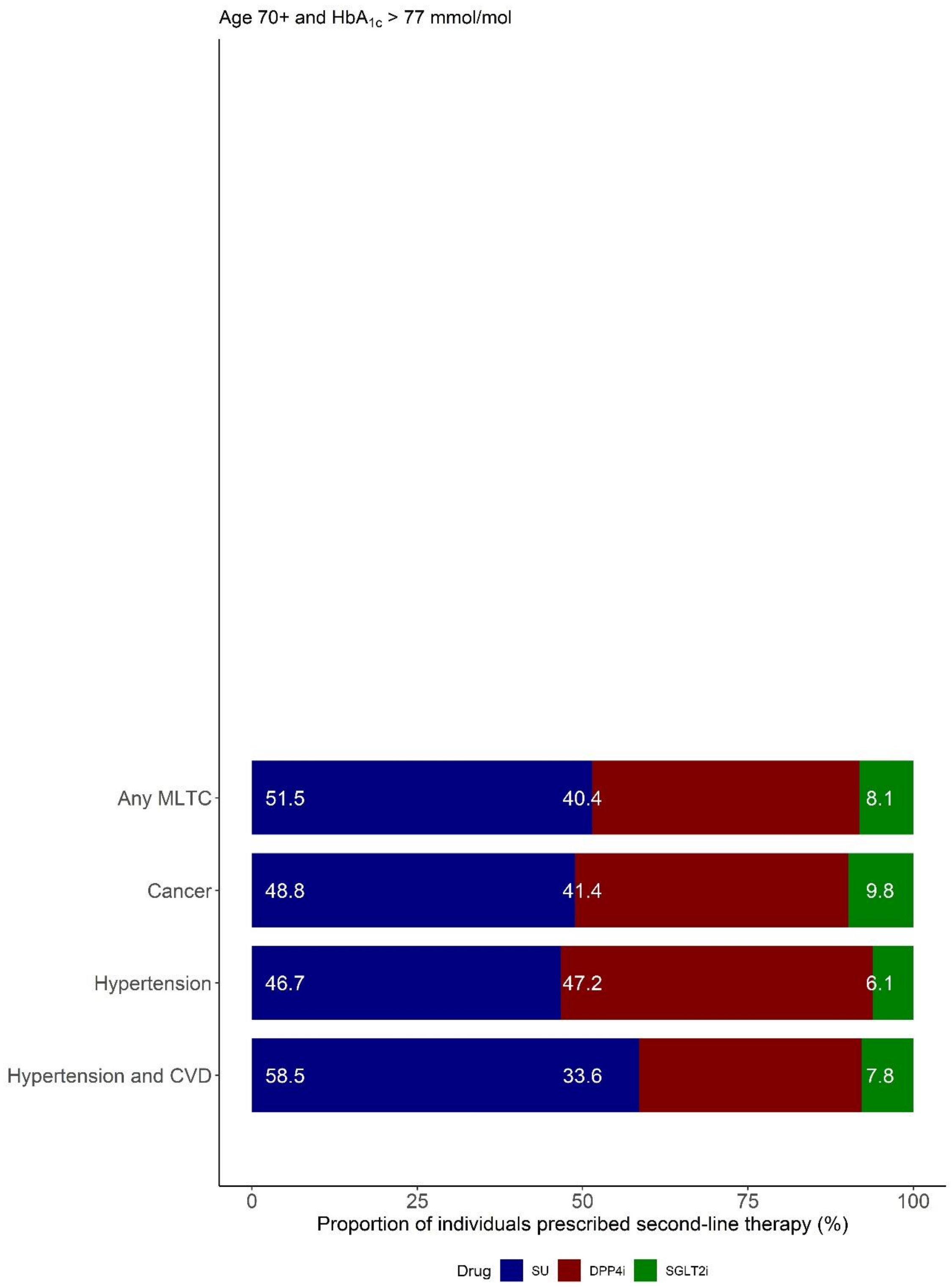

Age 70+ and HbA_{1c} > 77 mmol/mol
Any MLTC
Cancer
Hypertension
Hypertension and CVD
51.5
40.4
8.1
48.8
41.4
9.8
46.7
47.2
6.1
58.5
33.6
7.8
0
25
50
75
100
Proportion of individuals prescribed second-line therapy (%)
Drug
SU
DPP4i
SGLT2i

ESM Fig. 10: Mean level of rescaled baseline covariates according to the level of the instrumental variable

(a) **Tendency to prescribe DPP4i**

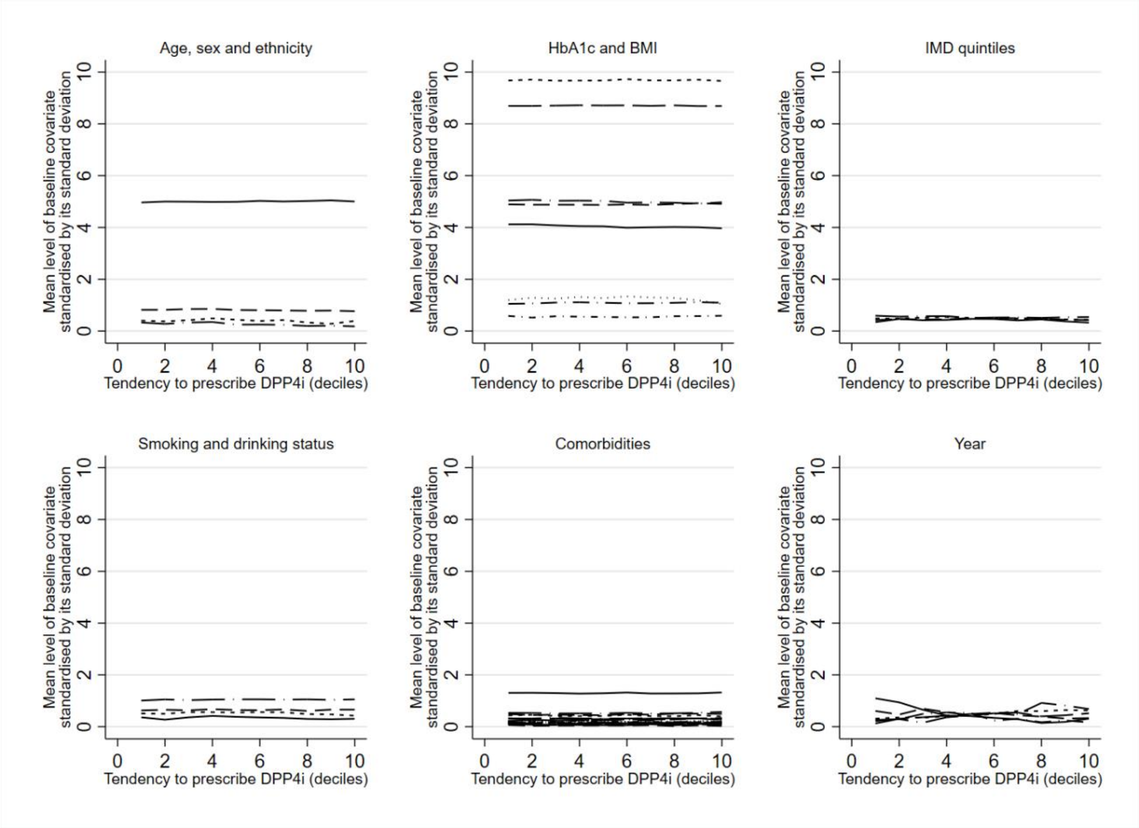

(b) **Tendency to prescribe SGLT2i**

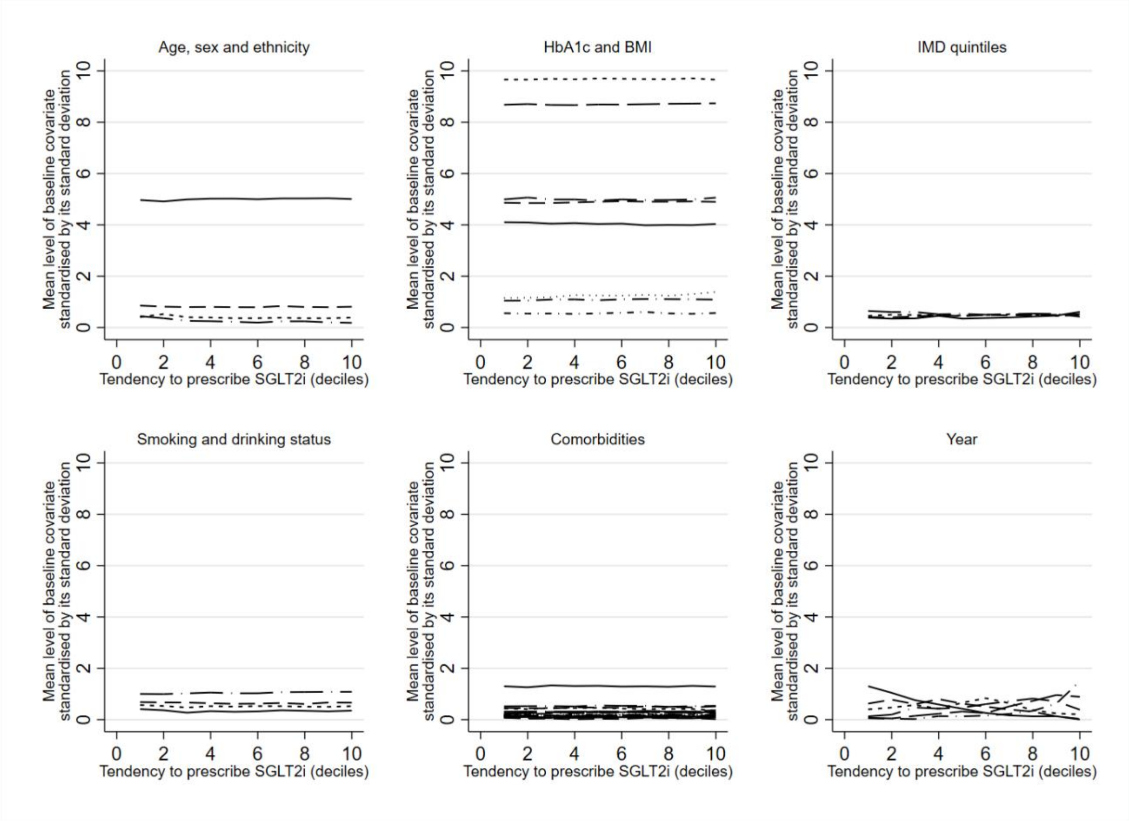

(c) Tendency to prescribe SU

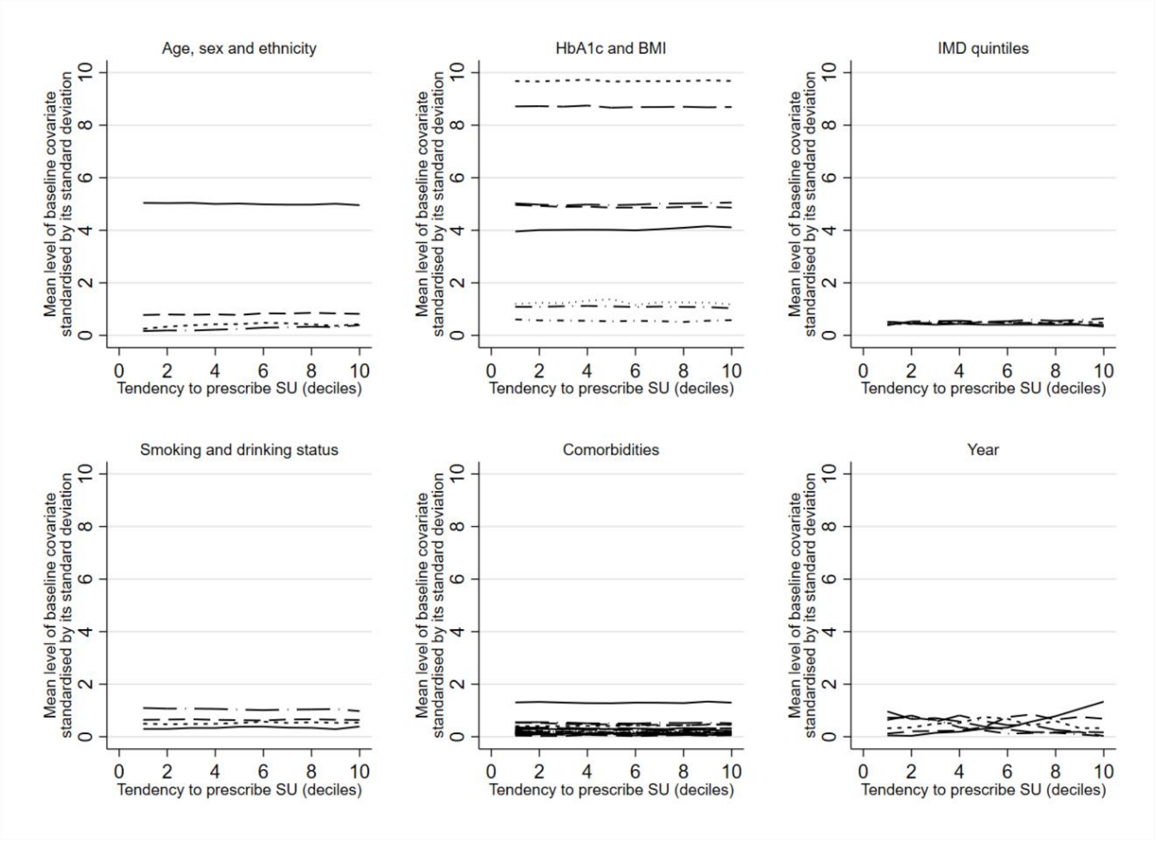

ESM Fig. 11: Crude (unadjusted) mean HbA_{1c} and change in mean HbA_{1c} from baseline to 0.5, 1, and 2-years follow-up for those who initiated second-line oral glucose-lowering treatment with one of sulfonylureas (SU), dipeptidyl peptidase-4 inhibitors (DPP4i), and sodium-glucose co-transporter 2 inhibitors (SGLT2i) all added to metformin monotherapy

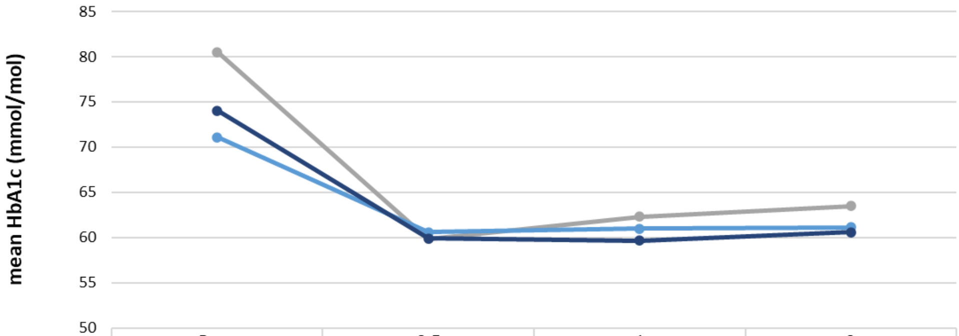

	Base	0.5	1	2
SU	80.5	59.87	62.32	63.47
DPP4i	71.11	60.6	60.99	61.13
SGLT2i	74.02	59.9	59.63	60.61

Year of follow-up and mean HbA1c (mmol/mol)

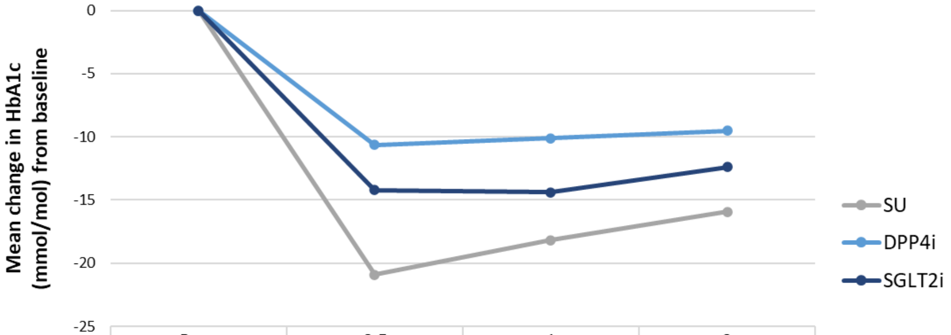

	Base	0.5	1	2
SU	0	-20.93	-18.18	-15.95
DPP4i	0	-10.64	-10.12	-9.52
SGLT2i	0	-14.21	-14.39	-12.39

Year of follow-up and mean change in HbA1c (mmol/mol) from baseline

ESM Fig. 12: Forest plot showing differences in the change in HbA_{1c} between baseline and 1-year follow-up for (i) DPP4i compared to SU, (ii) SGLT2 compared to SU, and (iii) SGLT2i compared to DPP4i for MLTC profile by HbA_{1c} subgroups for those aged 18 to 49 years.

(a)

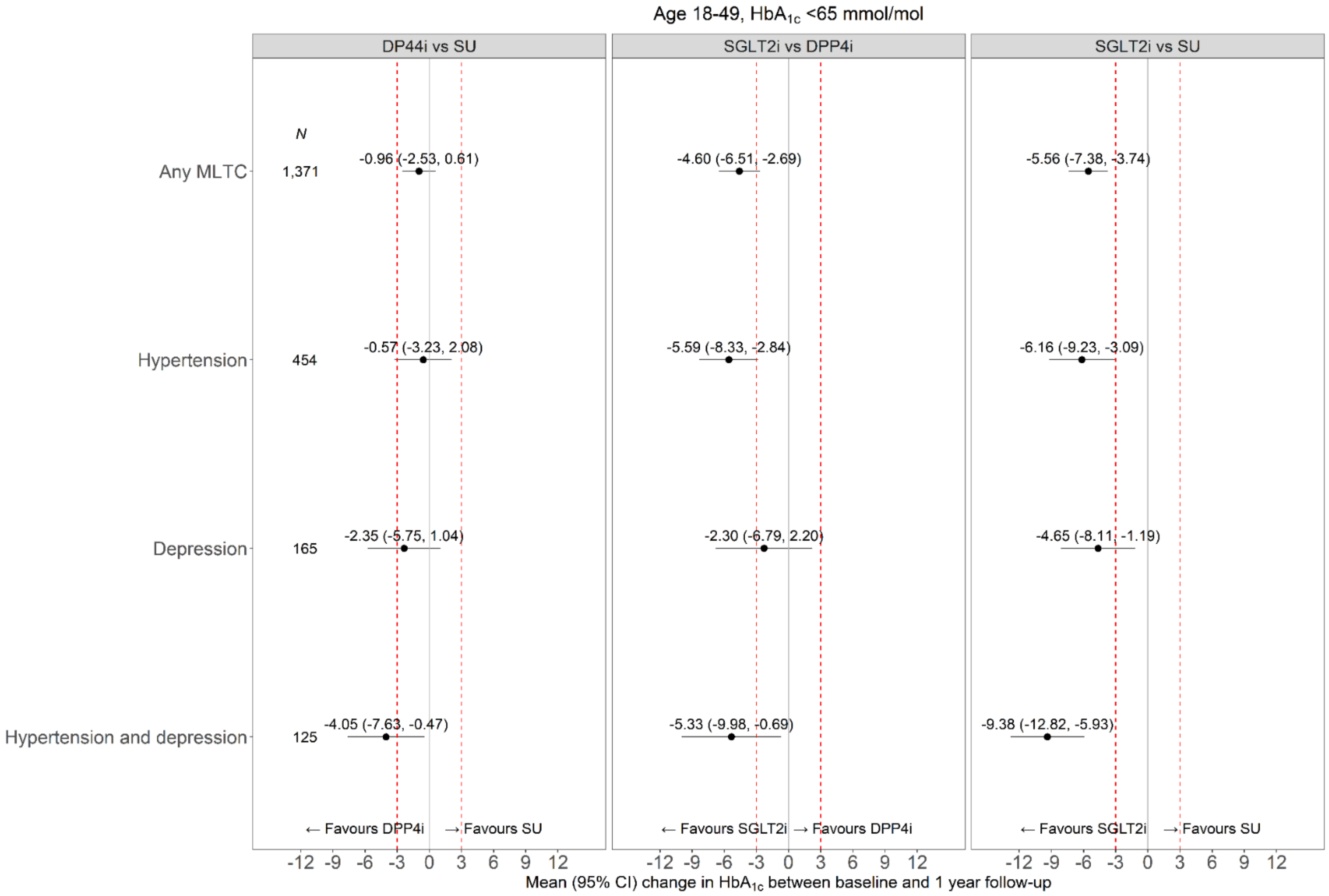

(b)

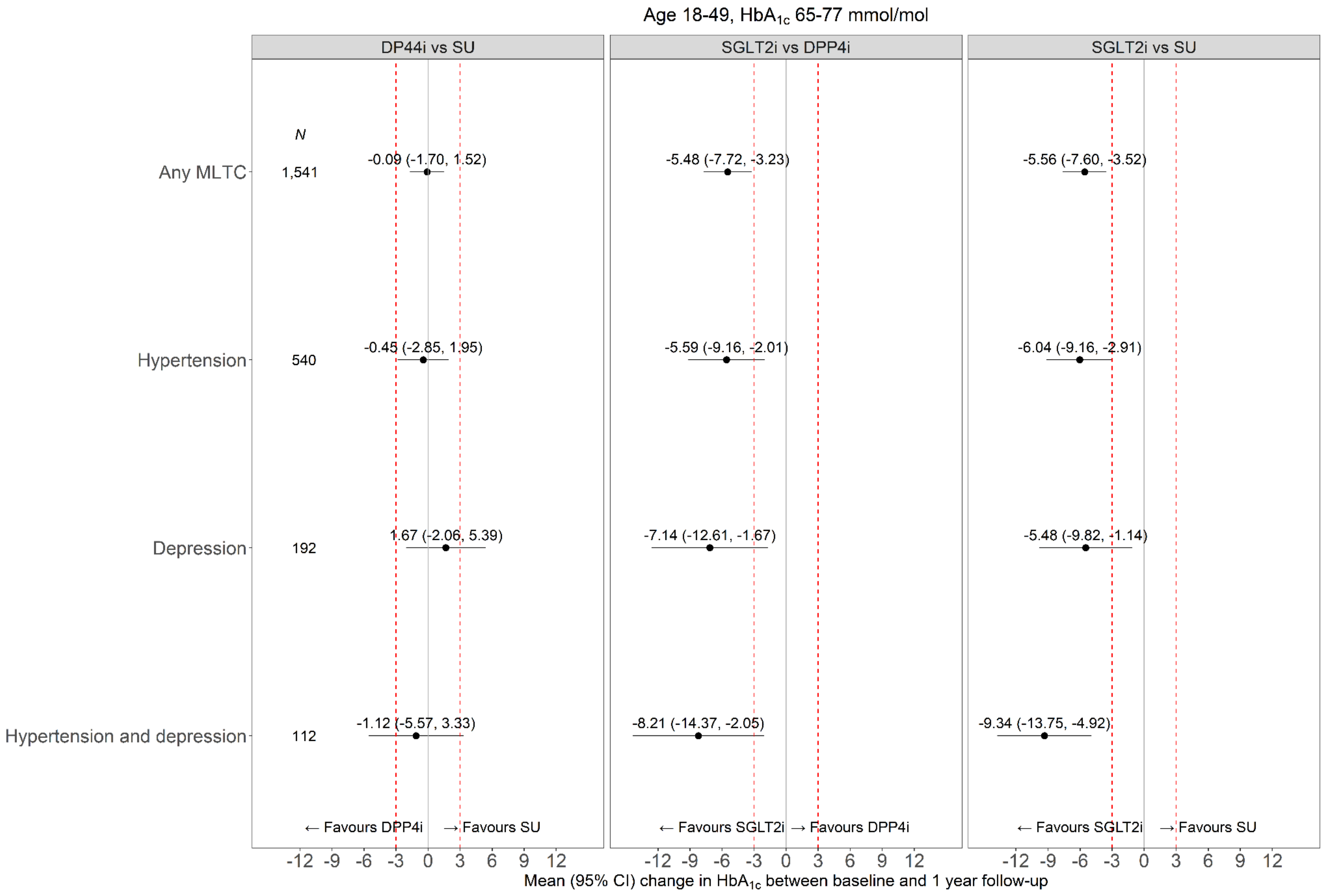

Age 18-49, HbA_{1c} 65-77 mmol/mol
DP44i vs SU
SGLT2i vs DPP4i
SGLT2i vs SU
N
Any MLTC
1,541
-0.09 (-1.70, 1.52)
-5.48 (-7.72, -3.23)
-5.56 (-7.60, -3.52)
Hypertension
540
-0.45 (-2.85, 1.95)
-5.59 (-9.16, -2.01)
-6.04 (-9.16, -2.91)
Depression
192
1.67 (-2.06, 5.39)
-7.14 (-12.61, -1.67)
-5.48 (-9.82, -1.14)
Hypertension and depression
112
-1.12 (-5.57, 3.33)
-8.21 (-14.37, -2.05)
-9.34 (-13.75, -4.92)
← Favours DPP4i → Favours SU
← Favours SGLT2i → Favours DPP4i
← Favours SGLT2i → Favours SU
-12 -9 -6 -3 0 3 6 9 12
Mean (95% CI) change in HbA_{1c} between baseline and 1 year follow-up

(c)

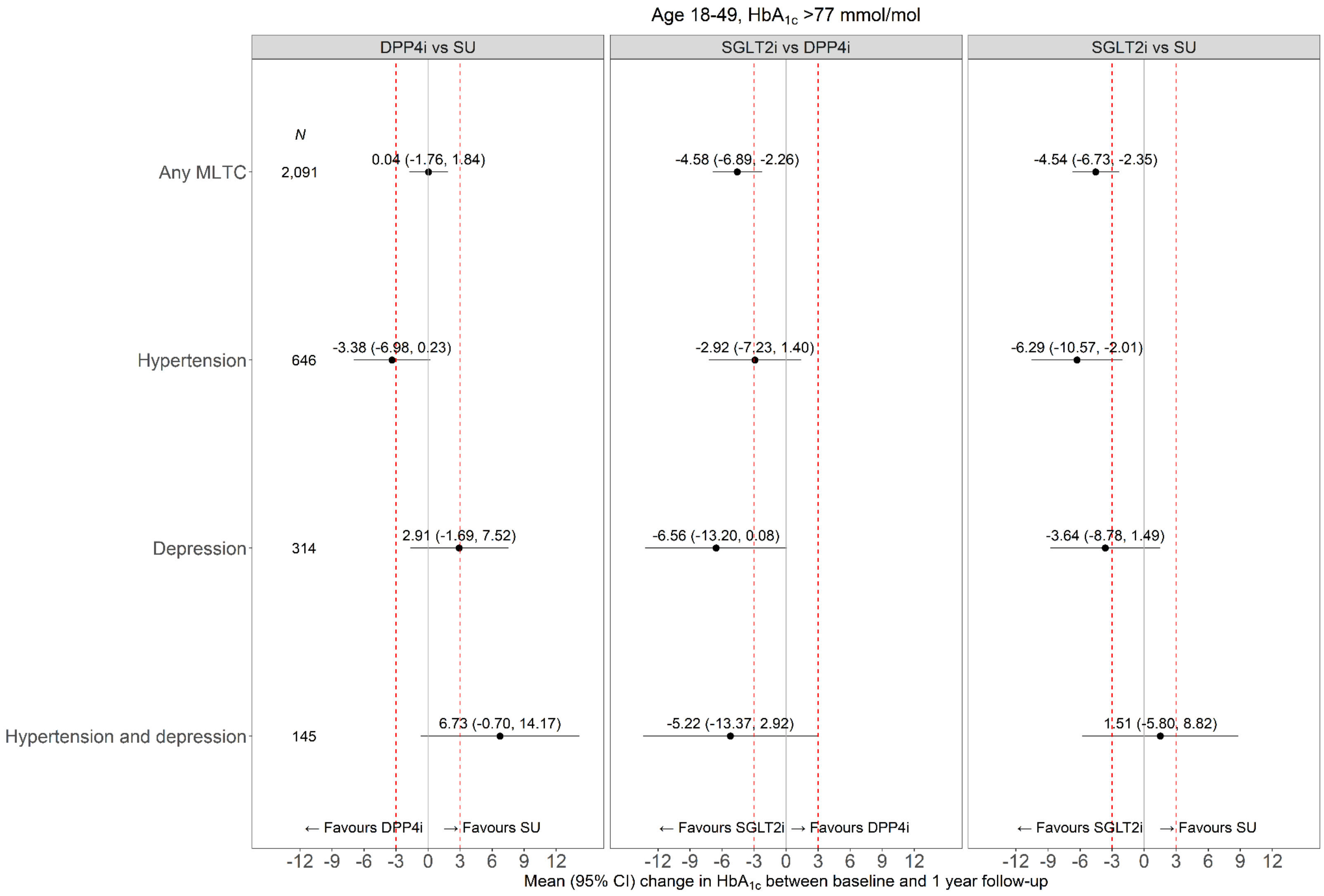

Age 18-49, HbA1c >77 mmol/mol
DPP4i vs SU
SGLT2i vs DPP4i
SGLT2i vs SU
N
Any MLTC
2,091
0.04 (-1.76, 1.84)
-4.58 (-6.89, -2.26)
-4.54 (-6.73, -2.35)
Hypertension
646
-3.38 (-6.98, 0.23)
-2.92 (-7.23, 1.40)
-6.29 (-10.57, -2.01)
Depression
314
2.91 (-1.69, 7.52)
-6.56 (-13.20, 0.08)
-3.64 (-8.78, 1.49)
Hypertension and depression
145
6.73 (-0.70, 14.17)
-5.22 (-13.37, 2.92)
1.51 (-5.80, 8.82)
← Favours DPP4i → Favours SU
← Favours SGLT2i → Favours DPP4i
← Favours SGLT2i → Favours SU
-12 -9 -6 -3 0 3 6 9 12
Mean (95% CI) change in HbA1c between baseline and 1 year follow-up

ESM Fig. 13: Forest plot showing differences in the change in HbA_{1c} between baseline and 1-year follow-up for (i) DPP4i compared to SU, (ii) SGLT2 compared to SU, and (iii) SGLT2i compared to DPP4i for MLTC profile by HbA_{1c} subgroups for those aged 50 to 69 years.

(a)

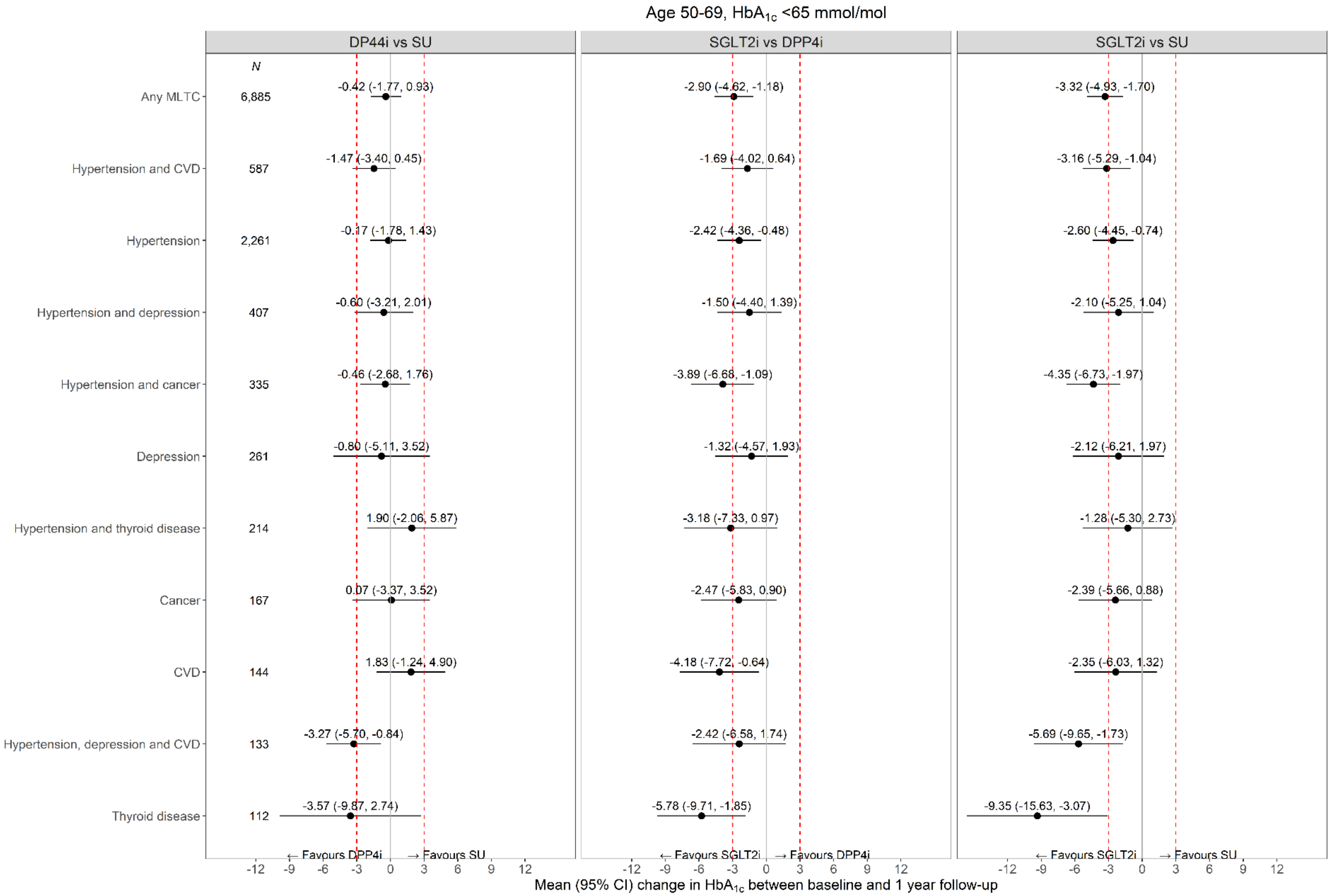

(b)

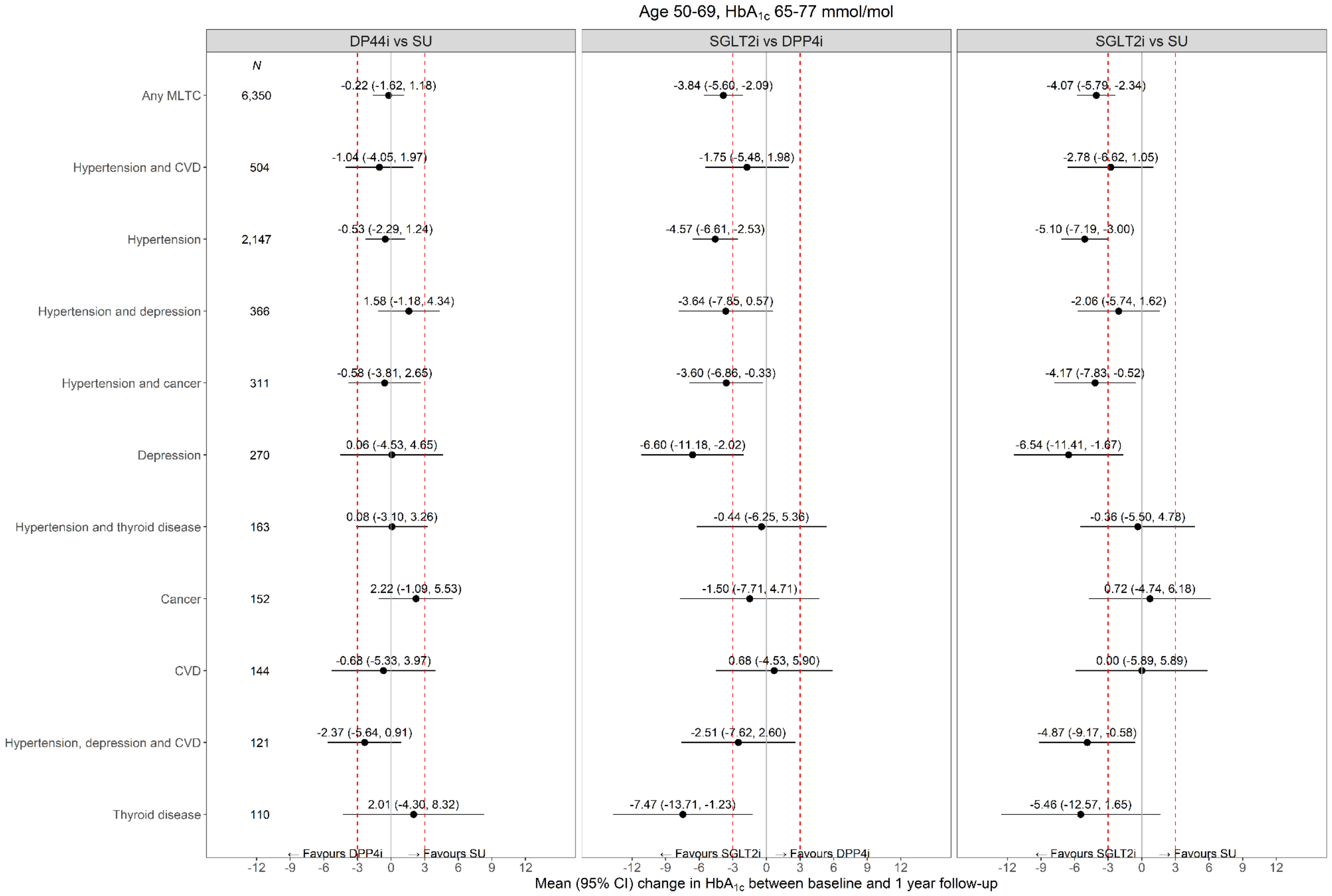

Age 50-69, HbA1c 65-77 mmol/mol
DP44i vs SU
SGLT2i vs DPP4i
SGLT2i vs SU
N
Any MLTC 6,350
Hypertension and CVD 504
Hypertension 2,147
Hypertension and depression 366
Hypertension and cancer 311
Depression 270
Hypertension and thyroid disease 163
Cancer 152
CVD 144
Hypertension, depression and CVD 121
Thyroid disease 110
-0.22 (-1.62, 1.18)
-1.04 (-4.05, 1.97)
-0.53 (-2.29, 1.24)
1.58 (-1.18, 4.34)
-0.58 (-3.81, 2.65)
0.06 (-4.53, 4.65)
0.08 (-3.10, 3.26)
2.22 (-1.09, 5.53)
-0.68 (-5.33, 3.97)
-2.37 (-5.64, 0.91)
2.01 (-4.30, 8.32)
-3.84 (-5.60, -2.09)
-1.75 (-5.48, 1.98)
-4.57 (-6.61, -2.53)
-3.64 (-7.85, 0.57)
-3.60 (-6.86, -0.33)
-6.60 (-11.18, -2.02)
-0.44 (-6.25, 5.36)
-1.50 (-7.71, 4.71)
0.68 (-4.53, 5.90)
-2.51 (-7.62, 2.60)
-7.47 (-13.71, -1.23)
-4.07 (-5.79, -2.34)
-2.78 (-6.62, 1.05)
-5.10 (-7.19, -3.00)
-2.06 (-5.74, 1.62)
-4.17 (-7.83, -0.52)
-6.54 (-11.41, -1.67)
-0.36 (-5.50, 4.78)
0.72 (-4.74, 6.18)
0.00 (-5.89, 5.89)
-4.87 (-9.17, -0.58)
-5.46 (-12.57, 1.65)
← Favours DPP4i → Favours SU
← Favours SGLT2i → Favours DPP4i
← Favours SGLT2i → Favours SU
-12 -9 -6 -3 0 3 6 9 12
Mean (95% CI) change in HbA1c between baseline and 1 year follow-up

(c)

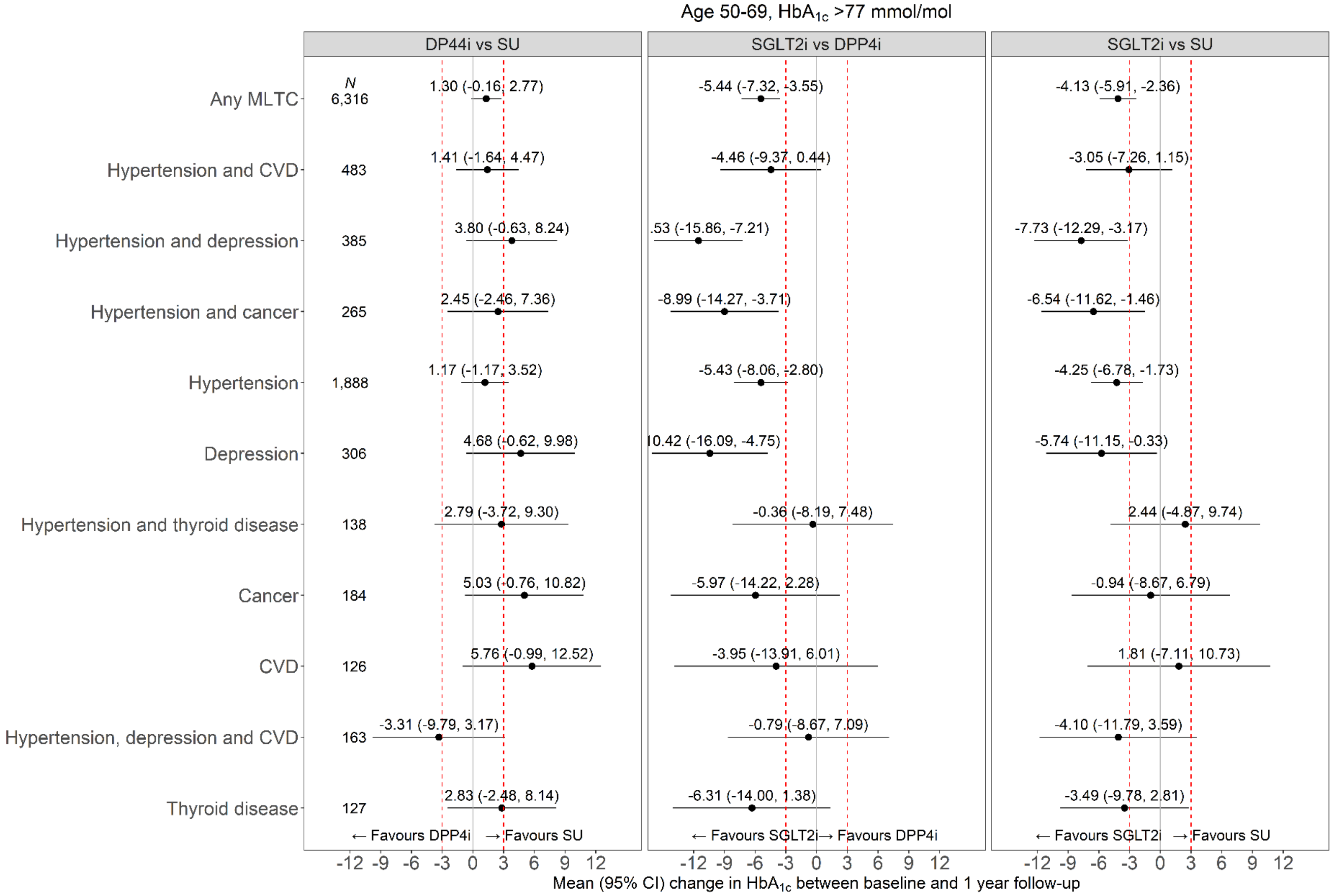

Age 50-69, HbA1c >77 mmol/mol
DP44i vs SU
SGLT2i vs DPP4i
SGLT2i vs SU
N
Any MLTC
6,316
1.30 (-0.16, 2.77)
-5.44 (-7.32, -3.55)
-4.13 (-5.91, -2.36)
Hypertension and CVD
483
1.41 (-1.64, 4.47)
-4.46 (-9.37, 0.44)
-3.05 (-7.26, 1.15)
Hypertension and depression
385
3.80 (-0.63, 8.24)
.53 (-15.86, -7.21)
-7.73 (-12.29, -3.17)
Hypertension and cancer
265
2.45 (-2.46, 7.36)
-8.99 (-14.27, -3.71)
-6.54 (-11.62, -1.46)
Hypertension
1,888
1.17 (-1.17, 3.52)
-5.43 (-8.06, -2.80)
-4.25 (-6.78, -1.73)
Depression
306
4.68 (-0.62, 9.98)
10.42 (-16.09, -4.75)
-5.74 (-11.15, -0.33)
Hypertension and thyroid disease
138
2.79 (-3.72, 9.30)
-0.36 (-8.19, 7.48)
2.44 (-4.87, 9.74)
Cancer
184
5.03 (-0.76, 10.82)
-5.97 (-14.22, 2.28)
-0.94 (-8.67, 6.79)
CVD
126
5.76 (-0.99, 12.52)
-3.95 (-13.91, 6.01)
1.81 (-7.11, 10.73)
Hypertension, depression and CVD
163
-3.31 (-9.79, 3.17)
-0.79 (-8.67, 7.09)
-4.10 (-11.79, 3.59)
Thyroid disease
127
2.83 (-2.48, 8.14)
-6.31 (-14.00, 1.38)
-3.49 (-9.78, 2.81)
← Favours DPP4i → Favours SU
← Favours SGLT2i → Favours DPP4i
← Favours SGLT2i → Favours SU
-12 -9 -6 -3 0 3 6 9 12
Mean (95% CI) change in HbA1c between baseline and 1 year follow-up

References

1. Wilkinson S, Williamson E, Pokrajac A, et al. Comparative effects of sulphonylureas, dipeptidyl peptidase-4 inhibitors and sodium-glucose co-transporter-2 inhibitors added to metformin monotherapy: a propensity-score matched cohort study in UK primary care. *Diabetes, Obesity and Metabolism*. 2020;22(5):847-856. doi:https://doi.org/10.1111/dom.13970
2. NHS England - About Us - Regional Teams. Web. NHS England. Accessed 28 June, 2023. https://www.england.nhs.uk/about/regional-area-teams/
3. Bhaskaran K, Forbes HJ, Douglas I, Leon DA, Smeeth L. Representativeness and optimal use of body mass index (BMI) in the UK Clinical Practice Research Datalink (CPRD). *BMJ Open*. 2013;3(9):e003389. doi:10.1136/bmjopen-2013-003389
4. Staiger D, Stock JH. Instrumental Variables Regression with Weak Instruments. *Econometrica*. 1997;65(3):557-586. doi:10.2307/2171753
5. Bidulka P, Lugo-Palacios DG, Carroll O, et al. Comparative effectiveness of second line oral antidiabetic treatments among people with type 2 diabetes mellitus: emulation of a target trial using routinely collected health data. *BMJ (Clinical research ed)*. 2024;385:e077097. doi:10.1136/bmj-2023-077097
6. StataCorp. Stata Statistical Software: Release 17. StataCorp LLC.
7. Basu A, Rathouz PJ. Estimating marginal and incremental effects on health outcomes using flexible link and variance function models. *Biostatistics*. Jan 2005;6(1):93-109. doi:10.1093/biostatistics/kxh020

8. Belloni, A., Chernozhukov, V. and Wei, Y., 2016. Post-selection inference for generalized linear models with many controls. *Journal of Business & Economic Statistics*, *34*(4), pp.606-619.
